# Structural Evolution of Layered Manganese Oxysulfides during Reversible Electrochemical Lithium Insertion and Copper Extrusion

**DOI:** 10.1021/acs.chemmater.1c00375

**Published:** 2021-05-24

**Authors:** Sunita Dey, Dongli Zeng, Paul Adamson, Jordi Cabana, Sylvio Indris, Jingyu Lu, Simon J. Clarke, Clare P. Grey

**Affiliations:** † Department of Chemistry, University of Cambridge, Lensfield Road, Cambridge CB2 1EW, U.K.; ‡ Department of Chemistry, State University of New York, Stony Brook, New York 11794-3400, United States; § Department of Chemistry, University of Oxford, Inorganic Chemistry Laboratory, South Parks Road, Oxford OX1 3QR, U.K.

## Abstract

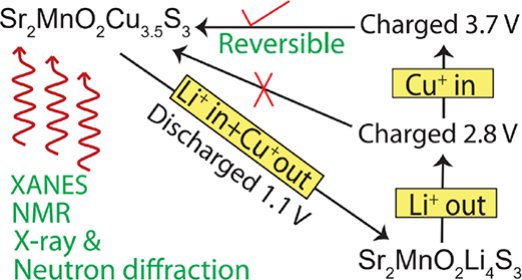

The electrochemical lithiation and delithiation of the layered oxysulfide Sr_2_MnO_2_Cu_4−δ_S_3_ has been investigated by using a combination of *in situ* powder X-ray diffraction and *ex situ* neutron powder diffraction, X-ray absorption and ^7^Li NMR spectroscopy, together with a range of electrochemical experiments. Sr_2_MnO_2_Cu_4−δ_S_3_ consists of [Sr_2_MnO_2_] perovskite-type cationic layers alternating with highly defective antifluorite-type [Cu_4−δ_S_3_] (δ ≈ 0.5) anionic layers. It undergoes a combined displacement/intercalation (CDI) mechanism on reaction with Li, where the inserted Li replaces Cu, forming Li_4_S_3_ slabs and Cu^+^ is reduced and extruded as metallic particles. For the initial 2–3% of the first discharge process, the vacant sites in the sulfide layer are filled by Li; Cu extrusion then accompanies further insertion of Li. Mn^2.5+^ is reduced to Mn^2+^ during the first half of the discharge. The overall charging process involves the removal of Li and re-insertion of Cu into the sulfide layers with re-oxidation of Mn^2+^ to Mn^2.5+^. However, due to the different diffusivities of Li and Cu, the processes operating on charge are quite different from those operating during the first discharge: charging to 2.75 V results in the removal of most of the Li, little reinsertion of Cu, and good capacity retention. A charge to 3.75 V is required to fully reinsert Cu, which results in significant changes to the sulfide sublattice during the following discharge and poor capacity retention. This detailed structure–property investigation will promote the design of new functional electrodes with improved device performance.

## 1 Introduction

Despite the deep penetration of Li-ion secondary batteries into the market, a need remains to continue to improve them through the optimization of currently available electrode materials and the discovery of new ones due to massive increases in market demands. Electrode materials can be classified into three types based on the mechanisms of (de)lithiation: intercalation, conversion, and displacement.^1^ The most widely used positive electrode (“cathode”) materials in commercial rechargeable lithium-ion batteries are intercalation compounds, such as layered LiCoO_2_, spinel-type LiMn_2_O_4_, olivine-type LiFePO_4_, and their substituted variants (in particular Ni-rich layered materials). Their electrochemical activity is enabled by the presence of one (or more) 3d transition metal redox centers that function as the charge reservoir and a stable framework that is capable of reversible lithium intercalation. These have intrinsic capacity limitations: lithium can only be inserted into the vacant sites in the framework structure and the available charge in these systems is limited by the amount and the extent to which the redox species can change valence. Often, only one electron per transition metal cation is available. This limitation is circumvented in conversion and displacement reactions, leading to a greater capacity. In conversion reactions, metal binary compounds M*_x_*X*_y_* (M = Mn–Cu, Sn, Ru, Mo, W, etc. and X = F, O, S, N, P etc.) are lithiated and the metals are reduced to their elemental state. The structure of the electrode material is completely reorganized, leading to metal nanoparticles embedded or surrounded in a Li*_n_*X salt.^1−5^ Displacement reactions are related to conversion reactions but usually some part of the framework is preserved during the (de)lithiation processes. Displacement reactions have been observed for several Cu–Sn and Cu–Sb intermetallic alloys that can function as the negative electrode materials in Li batteries^6−8^ and even in materials that can act as positive electrodes.^9−11^ A prominent example of the latter is Cu_2.33_V_4_O_11_,^10^ which has a structure consisting of [V_4_O_11_]*_n_* layers, and interlayer Cu cations (Cu^+^ and Cu^2+^). When it reacts with Li electrochemically, a reversible Li-driven process leading to the growth (on discharge) and disappearance (on charging) of Cu metal dendrites is observed, together with a concomitant exchange of Li for Cu in the interlayer sites. It shows a sustainable reversible capacity of over 250 mAh/g at a voltage around 2.7 V.^10^ Unfortunately, with a decrease in operating current (C/5 to C/10) and/or at a high applied voltage, the growth of Cu dendrites is exacerbated, leading to the quick failure of Cu_2.33_V_4_O_11_ activity.^12^ This system involves additional lithium intercalation that is charge-compensated by the V^5+^/V^4+^ redox couple, so the mechanism is described as a combination displacement/intercalation (CDI) reaction. Ag_2_V_4_O_11_, which is a cathode in primary lithium batteries, follows a similar mechanism.^9,13,14^

As Cu is known to be mobile in the spinel structure, the cation/anion mixed-valence thiospinels CuTi_2_S_4_ (Cu^+^Ti^3+^Ti^4+^(S^2–^)_4_) and CuCr_2_S_4_ (Cu^+^(Cr^3+^)_2_(S^2–^)_3_S^–^·^^) have also been examined.^15,16^ CuTi_2_S_4_ undergoes a CDI reaction in the initial stages of the first discharge (up to 2 Li inserted), where Li intercalation into the Ti_2_S_4_ framework is associated with extrusion of elemental Cu and complete reduction of Ti^4+^ to Ti^3+^. The electrochemical reaction of CuCr_2_S_4_ with Li initially also occurs through a CDI reaction, with the reduction of Cu and S^–^·^^, but Cu cannot be totally reduced and extruded (the end-of-discharge phase is Li_1.75_Cu_0.25_Cr_2_S_4_), and the reaction is only partially reversible. The differences between these two thiospinels have been ascribed to the higher mobility of Cu in CuTi_2_S_4_ than in CuCr_2_S_4_.^17^ A systematic study on a few other Cu–V–O and Cu–V–S systems has revealed that the dimensionality and flexibility of the framework structure, together with the Cu mobility in the material, play important roles in governing the CDI reactions.^17^ More recently, Li_2_Cu_2_O(SO_4_)_2_, has been investigated as a 4.7 V Li ion battery cathode. Although such a high voltage promotes electrolyte decomposition, the mechanistic study showed that Li_2_Cu_2_O(SO_4_)_2_ utilizes the Cu^2+/^Cu^3+^ couple at 4.7 V, while the reaction pathway follows either Li (de)insertion or complex displacement-conversion pathways depending on the voltage window.^18^

Another class of compounds that shows a displacement mechanism upon reaction with Li are the oxysulfides, *e.g*., Sr_2_MnO_2_Cu_2*m*–δ_S_*m*+1_ (*m* = 1, 2, and 3, δ ≈ 0.5).^19,20^ These materials consist of alternating perovskite-type [Sr_2_MnO_2_] sheets and antifluorite-type [Cu_2_S] slabs of varying thickness in which Cu^+^ ions occupy tetrahedral sites. Since δ is 0.5 in these compounds when synthesized using standard high temperature solid-state methods, the resulting vacant sites on the Cu^+^ sublattice are charge-compensated by mixed valency on the Mn sites (the average Mn oxidation state is +2.5). Previous studies have shown that Li can be both chemically^21^ and electrochemically^22,23^ inserted into the structure and elemental Cu extruded and that these reactions are reversible. Neutron powder diffraction (NPD) data for the chemically lithiated phases,^21^^7^Li NMR characterization,^22^ and scanning X-ray fluorescence imaging study^24^ of the electrochemically lithiated phases have confirmed that the inserted Li ions replace Cu in the tetrahedral sites of the sulfide layers, with extrusion of metallic Cu. The charge capacity of the Sr_2_MnO_2_Cu_2*m*–δ_S_*m*+1_ oxysulfides is proportional to the thickness of the sulfide layers.

The cyclability of these materials has been tested in Li batteries using a voltage window of 1.1 to 2.7 V, and the results have shown that (i) the retention of charge capacity in these oxysulfides largely depends on the thickness of the Cu_2_S layer, the *m* = 3 member shows the largest capacity; (ii) better performance in cycling but lower capacity was observed for the compounds with thinner sulfide layers (for *m* = 1); and (iii) the cycling performance of antifluorite-type Cu_2_S (regarded as the end member with infinite thickness of the Cu_2_S layer) under similar conditions was poor, meaning that the rigid perovskite-type [Sr_2_MnO_2_] layers in the structure seem to play a key role in providing structural stability of the framework upon Cu extrusion. The Cu^+^ vacancies in the sulfide layer, and the consequent variability in the Mn oxidation state, provide another contribution to the capacity on cycling. The electrochemical profiles of analogues Sr_2_MO_2_Cu_2_S_2_ with different transition metal cations (M = Ni or Co) are found to be distinctly different from the Sr_2_MnO_2_Cu_2*m*–δ_S_*m*+1_ systems. Co and Ni compounds contain many fewer vacancies in the Cu_2_S_2_ layers than the analogues with the more readily oxidized Mn cations, indicating that the composition of the oxide layer and/or the presence of vacancies in the copper sulfide slabs affects the lithiation processes.^23,25,26^ Recent work has also shown that anion redox involving sulfur can be important in controlling reaction pathways. For example, sulfides such as VS_4_^27,28^ and Li_2_FeS_2_^29^ undergo reversible ion intercalation/deintercalation reactions involving coupled S^2–^/S_2_^2–^ beside transition-metal redox couples.

**Figure 1:**
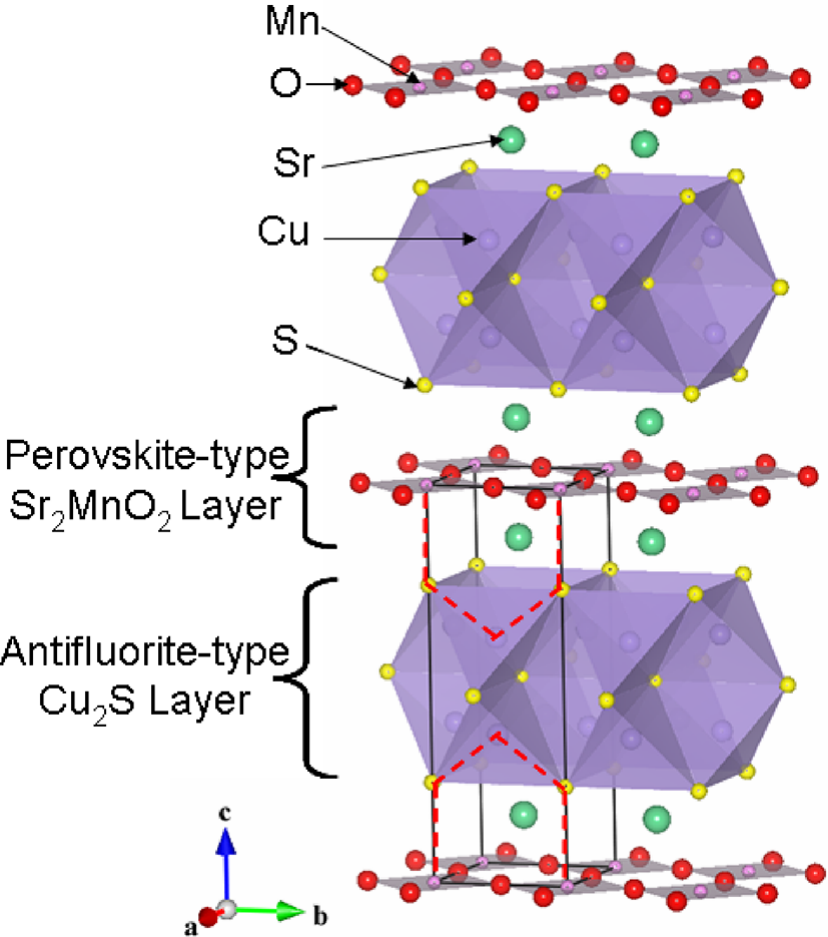
Crystal structure of Sr_2_MnO_2_Cu_3.5_S_3_ (MnCu(II)). The unit cell is indicated with black solid lines, the “MnO_2_” square planes and “CuS_4_” tetrahedra are shown in gray and purple, respectively. Sr: green, Mn: pink, O: red, Cu: blue, and S: yellow. The connectivities between the Cu and/or vacancy and two Mn (above or below) via bonds with S are highlighted with red dashed lines.

In this paper, we describe a detailed investigation of the electrochemical behavior of the *m =* 2 member of the Sr_2_MnO_2_Cu_2*m*–δ_S_*m*+1_ series, Sr_2_MnO_2_Cu_4−δ_S_3_ (denoted as MnCu(II) hereafter) (Figure 1) upon lithiation and delithiation. These materials were initially studied because of the potential of achieving materials that coupled some of the best properties of oxides and sulfides, namely, higher redox potentials and higher Li^+^ mobilities, respectively. MnCu(II) contains 3.5 of Cu^+^ ions per formula unit (Sr_2_MnO_2_Cu_3.5_S_3_), sufficient to occupy 7/8 of the tetrahedral sites in the sulfide layers.^20^ Previous studies have concluded that, upon lithiation, Cu^+^ and Mn^2.5+^ are reduced to Cu metal and Mn^2+^, respectively, so, in total, 4 Li^+^ ions are inserted into MnCu(II).^23,24^ However, the reactions accompanying electrochemical insertion of Li and extrusion of Cu have not been explored in detail. Charging up to 2.7 V (vs Li/Li^+^) is observed to be insufficient to extract all 4 Li^+^ ions; however, the details of the delithiation mechanism, when charged up to 2.7 V, has not been explored so far.^24^ Moreover, charging (as well as cyclability) above 2.7 V has not been explored to date; therefore, the device performance in the upper voltage range (>2.7 V) and the associated mechanisms are unknown.

This paper describes the structural changes during both the discharge and charge processes characterized by *in situ* powder X-ray diffraction (PXRD), *ex situ* neutron powder diffraction (NPD), and *ex situ*^7^Li NMR measurements. *Ex situ* X-ray absorption near-edge structure (XANES) measurements at the Cu, Mn, and S K edges have been analyzed to examine changes in oxidation states and local environments. Our results show that the Sr_2_MnO_2_ layer is far more than merely a structurally rigid “spacer” and is indeed redox-active, and we describe how the behavior is dependent on the charging voltage. This is important so as to understand their promising behavior, to assess mechanisms, and to guide the search of new functional electrode systems with improved performance.

## 2 Experimental Section

### 2.1 Sample Preparation

Sr_2_MnO_2_Cu_3.5_S_3_ was prepared as described previously^20^ by grinding together inside an argon-filled dry box, SrS, MnO_2_, Mn, CuO, and Cu_2_S in the ratio 2:0.25:0.75:1.5:1. Pellets of this mixture were heated in an alumina-lined evacuated silica ampoule at 1000 °C (ramp rate 10 °C/min) for 18 h. Electrochemically lithiated samples of MnCu(II) were recovered from Li batteries after reaching the desired state of charge (SOC) or depth of discharge (DOD). The electrode mixture consisting of 80 wt % of the oxysulfide, 10 wt % of acetylene black, and 10 wt % of polyvinylidene fluoride (PVDF) binder in *N*-methylpyrrolidone (NMP) was coated on a Al current collector. The batteries were assembled as coin cells (CR2032, Hohsen Corp.) in an argon-filled glove box. Each cell typically contains about 15 mg of oxysulfide, separated from a metallic Li (0.38 mm-thick foil, Aldrich, 99.9%) negative electrode by two pieces of Celgard separator (Celgard Inc., USA). A 1 M solution of LiPF_6_ in ethylene carbonate (EC):dimethyl carbonate (DMC) (1:1) was used as the electrolyte. The electrochemical experiments were carried out with a battery cycler in galvanostatic mode at a current density of 9.35 mA/g (a C/20 rate where C is defined as the theoretical capacity of the compound, 187 mAh/g). The cells were opened in the glove box, and the cathode films were washed with DMC and dried in a glove box atmosphere. The resulting samples were recovered in powder form and packed in rotors for *ex situ* NMR measurements and sealed as films with Kapton and Mylar tapes for *ex situ* PXRD and XANES experiments. Samples for powder neutron diffraction were obtained by scaling up the electrochemical syntheses.

Open-circuit potential data were collected by using the galvanostatic intermittent titration technique (GITT). Here, a constant current corresponding to a C/20 rate was applied for 4 h (during the first cycle between 1.1 and 3.75 V) or 1 h (during the second discharge from 2.75 to 1.1 V) followed by a rest period of 8 h for each step.

### 2.2 *In Situ* Powder X-ray Diffraction (PXRD)

*In situ* PXRD data were collected on beamline X18A at the National Synchrotron Light Source (NSLS) at Brookhaven National Laboratory (BNL), USA. The radiation wavelength was 0.9999 Å, and a step size of 0.02° 2θ was used. A specially designed cell (for *in situ* measurements)^30^ with Mylar windows was used to cycle the electrode films prepared as described above. Discharge to 1.1 V and subsequent charging to 2.8 V and partial discharging was accomplished over 48 h.

### 2.3 *Ex Situ* Neutron Powder Diffraction (NPD)

*Ex situ* neutron powder diffraction data were collected at 300 K for materials extracted from electrochemical cells on the POLARIS diffractometer at the ISIS facility, Rutherford Appleton Laboratory, UK. Batches of 0.2–0.4 g of the material were loaded into 6 mm diameter cylindrical vanadium cans sealed with indium gaskets inside an Ar-filled glove box to avoid any exposure to air and moisture. Data were collected for an integrated proton current at the target of between 1800 and 2500 μA h (dependent on the size of the sample). Rietveld refinement was carried out using the software package GSAS^31^ and its graphical user interface EXPGUI.^32^ The diffraction patterns collected have large backgrounds containing diffuse features, which are due to scattering from the acetylene black, added to improve conductivity. Due to the small sample volumes, Bragg peaks resulting from the vanadium sample cans were present in all the diffraction patterns.

### 2.4 X-ray Absorption Near-Edge Spectroscopy (XANES)

XANES spectra were collected on beamline X19A at the NSLS at BNL, USA. The measurements were performed in transmission (for Mn and Cu) or fluorescence (for S) mode using a Si (111) double-crystal monochromator detuned to 35–45% of its original intensity to eliminate the higher order harmonics. Energy calibration was carried out using the first inflection points in the spectra of Mn and Cu metal foil as references (Mn K-edge = 6539 eV, Cu K-edge = 8979 eV). The S K-edge XANES spectra were calibrated against the native sulfur K-edge at 2472 eV.

### 2.5 Solid-State Nuclear Magnetic Resonance (NMR) Spectroscopy

The ^7^Li magic-angle spinning (MAS) NMR experiments were performed at 77.8 MHz on a Chemagnetics CMX-200 spectrometer (B_0_ = 4.7 T) by using a double resonance 1.8 mm probe. Silicon nitride (Si_3_N_4_) rotors were used and spun at a speed of 38 kHz. All the spectra were acquired following a rotor-synchronized Hahn echo sequence (90°-τ-180°-τ-acquisition). The spectra were referenced to a standard 1 M LiCl solution at 0 ppm. π/2 pulses of 2 μs were typically used, with a delay time of 1 s.

## 3 Results

### 3.1 Electrochemistry

**Figure 2:**
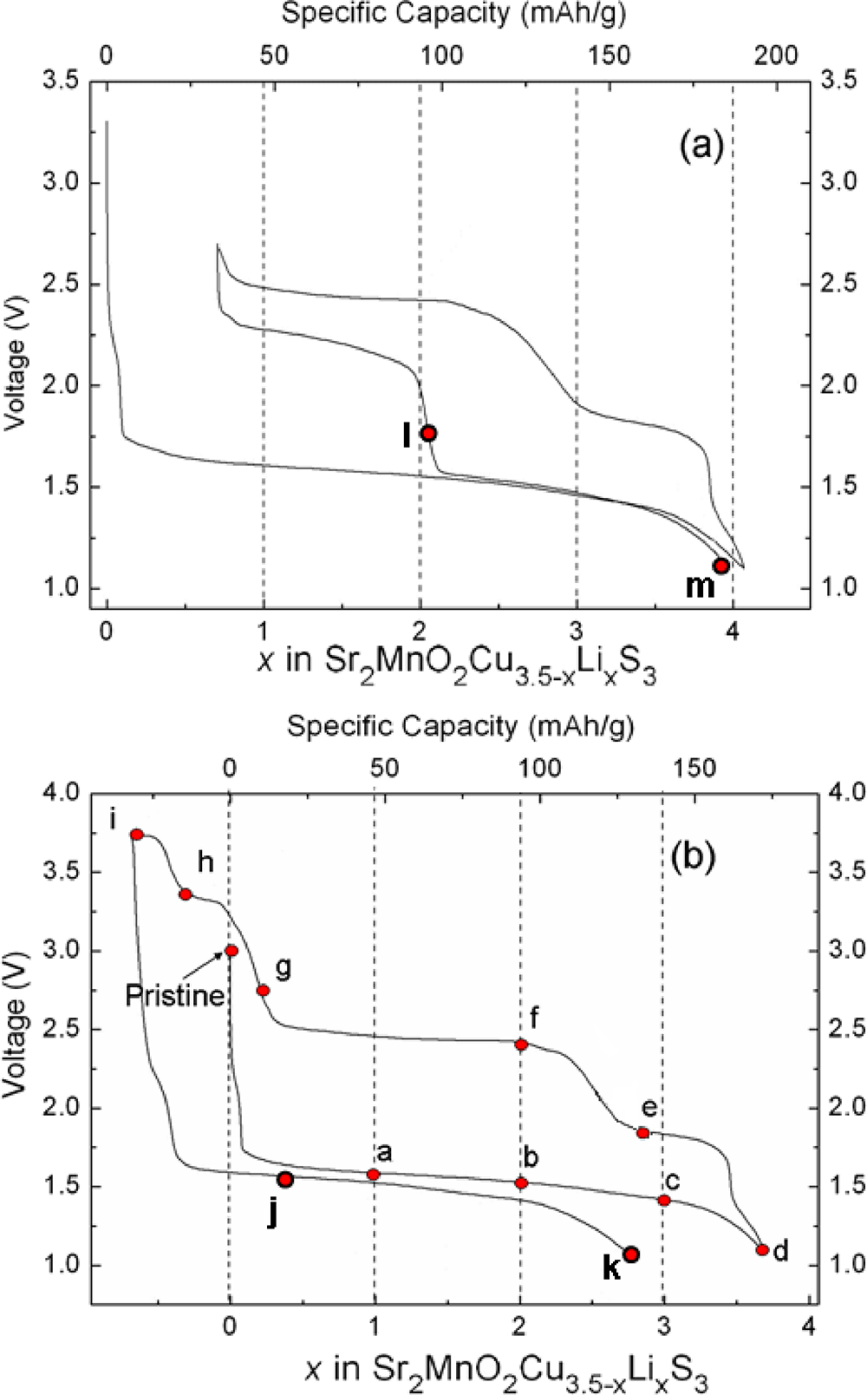
Plot of the voltage vs Li content and specific capacity of lithium batteries with MnCu(II) as the positive electrodes, cycled at a C/20 rate. The curves shown here include the complete first cycle (discharge and charge) and the second discharge for a battery cycled between (a) 1.1 and 2.75 V and (b) 1.1 and 3.75 V. The red points labeled at different stages of (dis)charge show the compositions of samples a–m prepared for the *ex situ* measurements. Summary of these processes are presented in Table S1, Supporting Information.

Figure 2 shows the voltage–composition curves of lithium batteries using MnCu(II) as positive electrodes and cycled between 1.1 and 2.75 V and between 1.1 and 3.75 V (see Table S1). After a process at around 2.1 to 2.2 V, which accounts for around 5 mAh/g (0.1 Li or ∼2.5%) of the capacity, the first discharge curve of MnCu(II) displays a long plateau at around 1.5 V, before reaching a capacity equivalent to about 4 mol of Li insertion per formula unit (187 mAh/g) at 1.1 V. The large capacity of this process suggests that it corresponds to Li^+^ ions completely replacing the Cu^+^ ions as well as occupying the vacant tetrahedral sites. Results from open-circuit voltage electrochemical measurements (GITT, Figure S1, Supporting Information) suggest an overpotential of around 0.2 V for the long plateau during the first discharge.

During the following charge to 2.75 V (Figure 2a), two distinct processes, at around 1.8 and 2.5 V, are resolved, which together account for 80% of the discharge capacity (*i.e*., there is 20% capacity loss after the first cycle when the system is charged to 2.75 V). The equilibrium potential for the “1.8 V” process is around 1.7 V (Figure S1a), very close to the potential of the second half of the 1.5 V process during the first discharge, suggesting that the species involved in the reaction may be the same.

The profile of the second discharge following charging to 2.75 V is different from that of the first discharge: two plateaus, at around 2.2 and 1.5 V are observed, each accounting for roughly half of the total capacity. The GITT results (see Figure S1b, Supporting Information) show that the equilibrium voltage of the “2.2 V” discharge process and the “2.5 V” charge process are similar. The two plateaus at 2.2 and 1.5 V are observed during subsequent cycles, a sign of good cyclability, and little loss of capacity is seen beyond that lost between the first and second discharges within the voltage window of 1.1–2.7 V.^22^ Furthermore, these two processes are similar to the two plateaus at around 2.1 and 1.7 V observed for CuS during the first discharge in a Li battery,^33,34^ except the capacity corresponding to each plateau is much smaller than that observed for CuS due to the much lower formula weight of CuS.

In contrast, when a battery was charged to 3.75 V, a voltage that is higher than that required to oxidize Cu^0^ (∼3.56 V),^35^ an additional capacity of approximately 30 mAh/g, was observed in Figure 2b. This extra capacity could be due to the oxidation of Cu^0^ remaining during earlier in the charge process to Cu^+^, which could either displace the remaining Li in the structure and/or migrate into the electrolyte. These hypotheses will be discussed below. A similar process was described earlier for the Li*_x_*CuTi_2_S_4_ system: capacities observed at 3.75 V were associated with the re-insertion of residual Cu into the structure that still remained in the electrode after charging at lower potentials and the removal of all of the Li, according to PXRD results.^15^

The cutoff voltage for the first charge clearly has an effect on the subsequent second discharge. When the first charge goes to 3.75 V, the second discharge curve closely follows that obtained for the first discharge, suggesting that the pristine structure of the oxysulfide is largely restored on charging the system to 3.75 V. The overall capacity is about 8% lower during the second discharge than the first. The drop in capacity on the second discharge is nearly 20% when the first charge is to just 2.75 V. However, the cycling performance of MnCu(II) using the larger voltage window (1.1–3.75 V) is poorer than when the 1.1–2.75 V window is used, and the capacity fades quickly to less than 50 mAh/g after 10 cycles (Figure S2).

*In situ* PXRD, *ex situ* PXRD and NPD, XANES, and NMR experiments were carried out to understand the processes occurring during Li insertion/extraction. The *ex situ* analyses were performed on samples extracted from batteries at different states, which are marked with red dots and labeled a–m in Figure 2.

### 3.2 X-ray and Neutron Powder Diffraction Measurements

**Figure 3:**
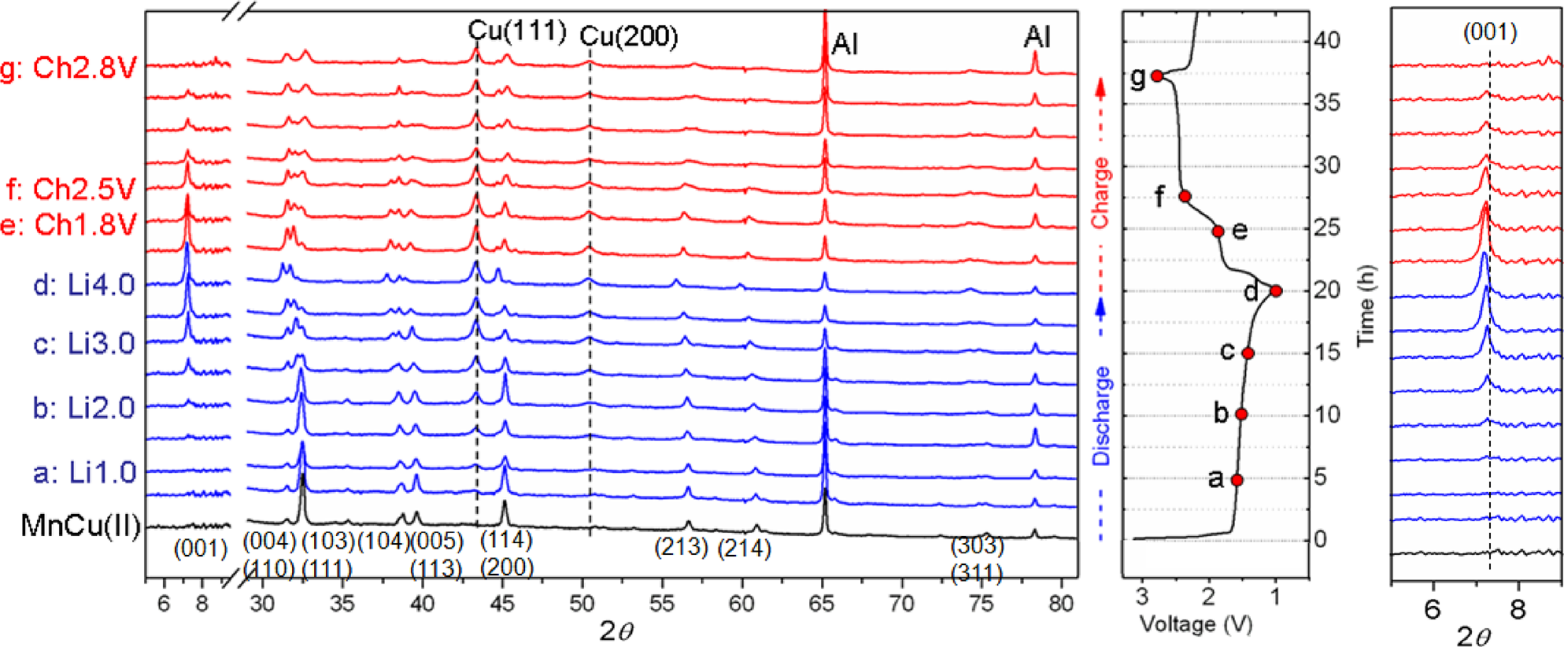
Representative *in situ* PXRD patterns (left panel) and corresponding electrochemical profile (middle panel) for a Li/MnCu(II) cell cycled between 1.1 and 2.8 V at a C/20 rate. The 2θ values have been converted to those corresponding to CuK_α_ (*i.e.*, a wavelength of 1.5418 Å)*.* Selected compositions at different depths/states of discharge/charge are marked with letters a–g in the electrochemical profile (using the same letter key as for *ex situ* samples), and the corresponding Li contents or voltage values are indicated on the XRD patterns. The reflections are indexed for the pristine MnCu(II) phase. Reflections due to Cu metal are indicated with dashed lines and are also indexed. The reflections from the Al foil in the *in situ* cell are marked. The right panel is a magnification of the region between 5° and 9° 2θ; the dashed line is a guide to the eye showing the highest 2θ angle reached by the (001) reflection of the oxysulfide.

*In situ* PXRD was used to probe the evolution of the oxysulfide phase(s) and extruded copper during the first discharge and the subsequent charge to 2.8 V (Figure 3). Separate samples were prepared at points in the first cycle, in the second discharge from 2.8 V, and also when the first charge was continued to 3.75 V in order to carry out *ex situ* PXRD (Figure 5) and NPD (Figure 6) measurements. Quantitative analysis of these patterns using Rietveld refinement (Figure S3) was used to investigate the phases and their compositions present during the discharge and charging processes. The refinement of Sr_2_MnO_2_Cu_3.5_S_3_ at the start of the discharge and the other oxysulfide phases identified are given in Table S2 in the Supporting Information.

#### 3.2.1 First Discharge

During the first discharge, reflections from elemental Cu become evident in the patterns [2θ = 43° (111) and 51° (200)] as indicated in Figure 3. The reflections due to the oxysulfide phase also change their intensities and positions as the phase becomes lithiated and copper is extruded. The analysis indicates that only one oxysulfide phase (which we designate OS-I) is present during the entire first discharge process to 1.1 V.

The reflections from elemental Cu initially appear weak and broad. The point where about 0.2 Li per mole of Sr_2_MnO_2_Cu_3.5_S_3_ have been inserted the most intense Cu reflection ((111) reflection) appears as a broad peak but is much narrower and intense by the time 0.5 moles of Li have been inserted per mole of Sr_2_MnO_2_Cu_3.5_S_3_. This is depicted in Figure S4, which shows the Rietveld fits in the early part of the discharge up to point a in the profile (Figure 2 and Figure 3). Since there are initially 0.5 moles of vacancies per mole of Sr_2_MnO_2_Cu_3.5_S_3_, the diffraction data show that Cu extrusion starts to occur well before all these vacancies are filled. The short process at 2.2 V on discharge appears to be associated with pure insertion of 0.1 Li at the start of the discharge, but the signal to noise ratio is not sufficient to exclude the possibility that Cu extrusion may also be occurring at this point. Along with the appearance of the reflections due to the formation of Cu metal, the reflections due to the oxysulfide phase change their intensities. In particular, the (001) reflection at 2θ = 7.7° gains intensity. This reflection happens to have negligible intensity in Sr_2_MnO_2_Cu_3.5_S_3_ (*i.e.*, the magnitude of the structure factor is coincidentally close to zero because of the relative phases of the scattering contributions of all the atoms) but acquires intensity as Cu is replaced by Li. This is because the transformation is topotactic (*i.e.*, the arrangement of scattering objects is unchanged), but the substitution of the strongly scattering Cu^+^ by the comparatively weakly scattering Li^+^ results in a significant increase in the magnitude of the structure factor for this reflection and hence an increase in the intensity (more details can be found in ref (21)).

**Figure 4:**
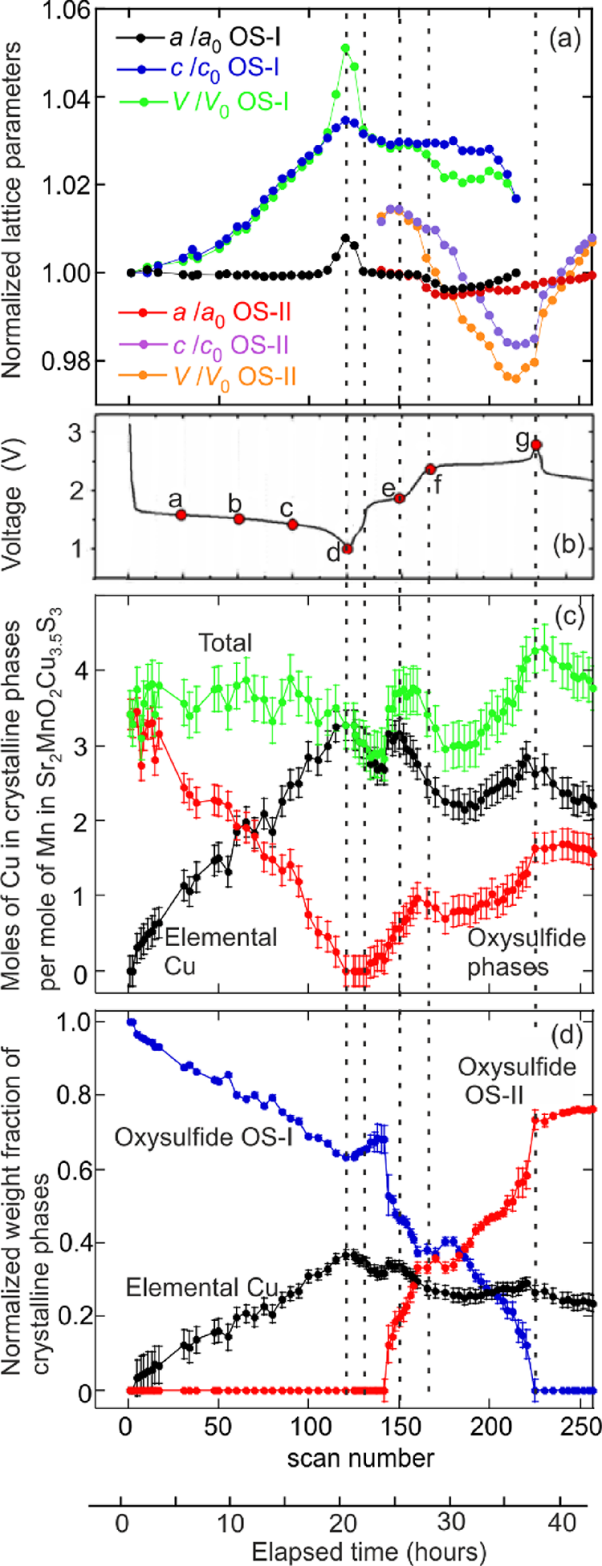
Results of quantitative analysis of the *in situ* PXRD data obtained during the first discharge followed by charging to 2.8 V (see Figure S3, Supporting Information for selected Rietveld fits). Panel (a) shows the evolution of the lattice parameters and unit cell volume of the oxysulfide phases OS-I and OS-II. The values have been normalized to those of OS-I (*i.e.*, Sr_2_MnO_2_Cu_3.5_S_3_) at the start of the discharge. Panel (c) shows the distribution of Cu amongst the crystalline phases (oxysulfides vs elemental Cu). Panel (d) shows the evolution of the weight fraction of the crystalline phases during the cycle. The dotted lines are guides to the eye.

The *in situ* data were modeled by using a single oxysulfide phase (OS-I) and modeling the composition by making the assumption that the Cu/Li sites are fully occupied and refining the Cu:Li ratio (Figure 4). After full discharge to 1.1 V, the lattice parameters show excellent quantitative agreement with those obtained for fully lithiated material obtained by reacting Sr_2_MnO_2_Cu_3.5_S_3_ with *n*-BuLi^21^ and, in the refined model, the scattering on the tetrahedral sites in the sulfide layer corresponds to full occupancy by Li. The evolution of the lattice parameters during discharge and the subsequent charge (Figure 4) shows that the previously documented ∼1.0% increase in the basal lattice parameter *a* on lithiation occurs almost wholly during the final 10–15% of the lithiation process;^21^ this, coupled with the much larger, but steadier increase in *c*, which occurs over the whole of the discharge, leads to a sharp increase in the cell volume at the end of the discharge. At this point in the discharge, a sample composition of Sr_2_MnO_2_Cu_0.00(3)_Li_4.00(3)_S_3_ and Cu in a 1:3.4(1) mole ratio has been obtained in the refinements (see Table S2). Despite the poor counting statistics of the data and the intrinsic difficulty of disentangling correlated parameters in the Rietveld refinement (notably between site occupancy factors and atomic displacement parameters), the refined composition of OS-I and the refinement of the phase fractions of OS-I and elemental Cu show that Cu is gradually replaced by Li in OS-I during discharge and that a fully lithiated material is obtained at the end of the discharge. The refinements show that the vast majority of the elemental Cu is present as a crystalline phase, and the apparent total Cu content in the Cu + oxysulfide crystalline phases remain constant within the uncertainty during the first discharge (Figure 4c).

**Figure 5:**
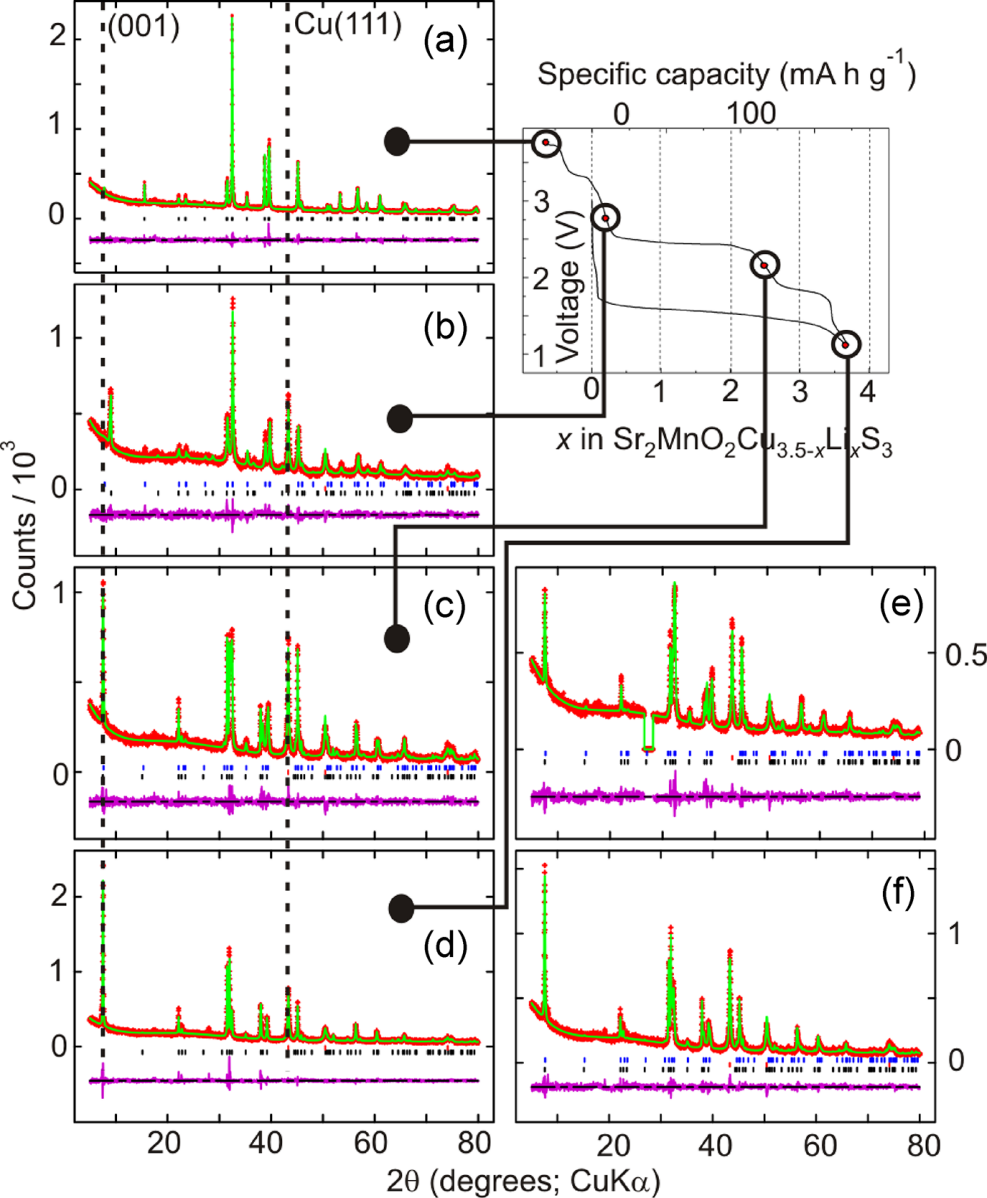
Rietveld refinements against laboratory PXRD data of samples extracted from batteries during the first charge (diffractograms (a)–(d)) at the voltages shown and during the second discharge after charging to 2.8 V (diffractograms (e) and (f)). Diffractograms (e) and (f) correspond to the 2.2 and 1.1 V discharged states during the second discharge, respectively. The dotted lines show the position of the most intense (111) reflection from elemental Cu (which is entirely absent when the battery is charged up to 3.75 V) and the position of the (001) reflection in Cu or Li containing oxysulfide phases. The position of this (001) reflection shifts to a much higher 2θ for a minority phase (OS-III) present in diffractogram (b), and this phase is tentatively modeled as a “collapsed” oxysulfide phase completely devoid of cations in the sulfide layer. Corresponding fits to NPD data for the same samples are shown in Figure 6.

**Figure 6:**
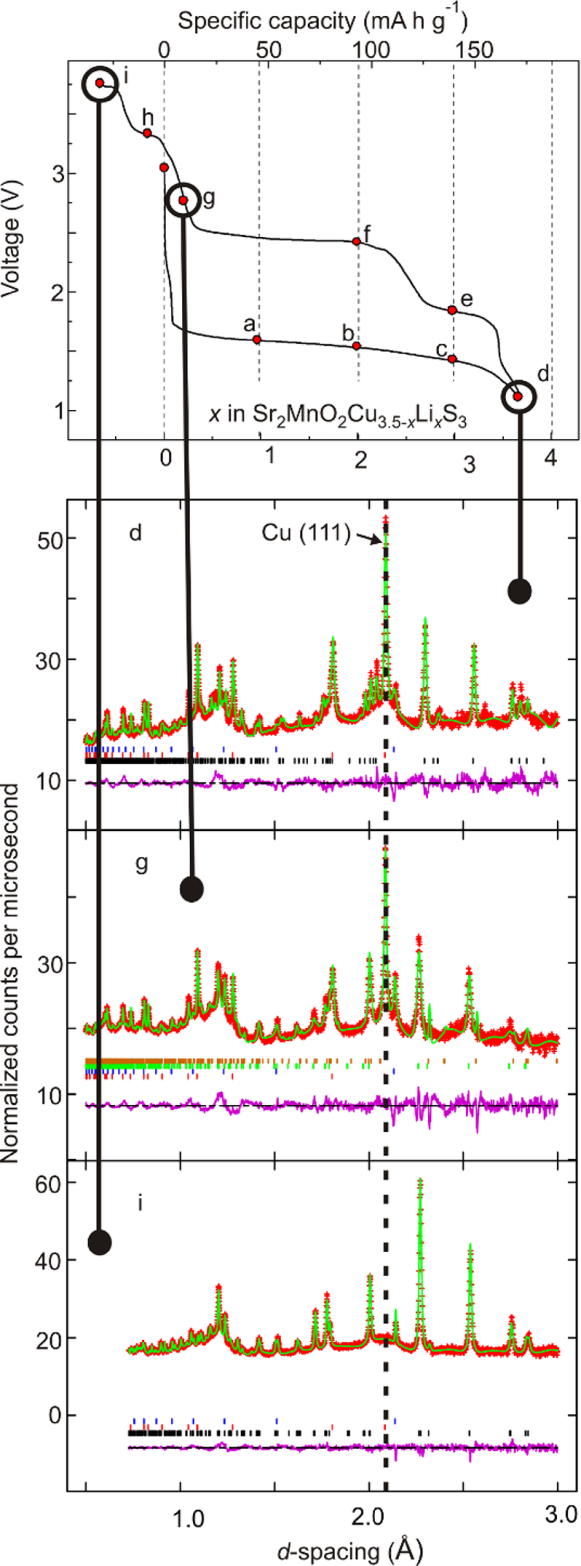
Rietveld analysis of NPD data (POLARIS, ISIS facility) of samples extracted from batteries that were fully discharged (d), charged to 2.8 V (g), and charged to 3.7 V (i). The dotted line is a guide to the eye, which shows that the strongest (111) peak due to elemental Cu is not discernable in the sample charged to 3.7 V.

For comparison, a larger sample of a material discharged to 1.1 V was also examined with laboratory PXRD and NPD data (Figure 5 and Figure 6) and shows a smaller *a* lattice parameter than the *in situ* discharged sample (Table 1). Refinement suggests the composition was Sr_2_MnO_2_Cu_0.4(1)_Li_3.6(1)_S_3_ and elemental Cu in a 1: 2.9(1) mole ratio, which means that the fully discharged state had not quite been reached. The lower overall lithiation is likely a consequence of scaling up the electrochemical synthesis.

**Table 1:**
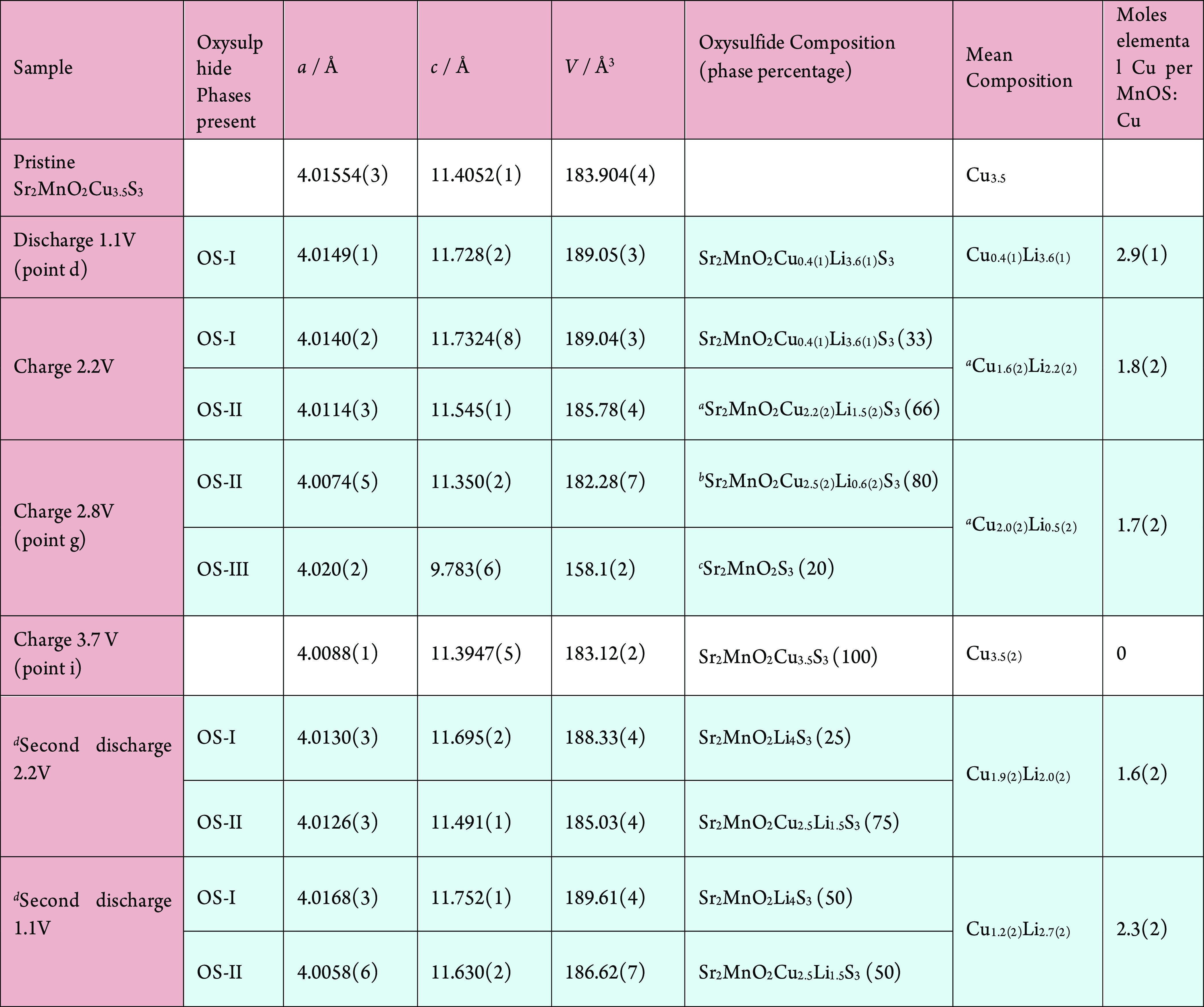
Refined Lattice Parameters, Compositions, and Phase Fractions for *Ex Situ* PXRD Refinements

a Overall Li content deduced from point on charge profile.

b OS-II phase assumed to contain remaining Li.

c Poorly characterized minority phase.

d *S*econd discharge after charging to 2.8 V.

#### 3.2.2 First Charge

The phase behavior during charging is complex. The *in situ* PXRD measurements were made on charging up to 2.8 V. At the start of the charge, the basal lattice parameter and the cell volume of the single oxysulfide phase (OS-I) decrease rapidly (Figure 4a), presumably as lithium is removed from the lithiated phase. The end of the sharp decrease in cell volume corresponds to the start of the 1.8 V charging process. At about the mid-point of the 1.8 V process, the data are best modeled by including a second oxysulfide phase (OS-II), which was not observed at any point during the first discharge. The analysis in the multiphase region was carried out by refining the compositions (*i.e*., the scattering present on the metal sites in the sulfide layer) as well as the phase fractions of the two oxysulfide phases (OS-I and OS-II) and elemental Cu. The refinements showed that on charging the OS-I phase has only very weakly scattering elements on the tetrahedral sites in the sulfide layer (*i.e.*, as Li is removed, very little Cu (less than 0.5 moles per mole of Mn) is reinserted into this phase). In contrast, the OS-II phase is relatively Cu-rich, with the scattering on the tetrahedral sites in the sulfide layer corresponding to about 1.8(3) moles of Cu per mole of Mn (about half of the amount in Sr_2_MnO_2_Cu_3.5_S_3_). The refined weight fraction of this Cu-containing OS-II phase increases progressively at the expense of OS-I as charging proceeds. Formation of this OS-II phase upon charge provides an explanation for the asymmetry observed in the voltage trace during the two processes; its origin lies on the fundamentally different mechanisms of reaction that the system follows, depending on whether lithium or copper is inserted or removed. The refined structure of the OS-II phase obtained from the *in situ* refinements after charging to 2.75 V where it becomes the only oxysulfide phase present is given in Table S2 in the Supporting Information.

The composition of the sample changes rapidly in between the end of the 1.8 V process (point e) to the start of the 2.5 V process (point f). From the fully discharged state to this point, the amount of elemental Cu in the sample diminishes and the weight fractions of the Cu-rich OS-II phase and the OS-I phase become approximately equal. To explore this further, *ex situ* diffraction measurements were carried out on a separate sample charged to a point midway between points e and f, corresponding to the region where the composition was changing most rapidly (Figure 5c). Analysis of this *ex situ* sample showed that a mixture of OS-I and OS-II was present, as in the *in situ* analysis, although OS-II constituted about two-thirds of the oxysulfide phases present, while in the *in situ* analysis, it constituted about one-half of the oxysulfide phases present. Considering that the *ex situ* and *in situ* samples were prepared separately under slightly different conditions and at different scales and that we cannot rule out evolution of the *ex situ* sample after removal from the cell, these results are comparable. At this point in the charge profile, it is estimated that the oxysulfide phases contain about 2.2 moles of Li per mole of Mn, on the basis of the electrochemistry. From the *ex situ* refinements, OS-I had lattice parameters very similar to those at the fully discharged (*i.e.*, fully lithiated) state and constituted about one-third of the oxysulfide phases present; refinement suggested a composition Sr_2_MnO_2_Li_3.6(1)_Cu_0.4(1)_S_3_ assuming full site occupancy in the sulfide layers, which is consistent with the composition of this phase obtained from the *in situ* refinements. OS-II, constituting about two-thirds of the oxysulfide phases present, had much smaller lattice parameters, but they were still larger than those of the parent Sr_2_MnO_2_Cu_3.5_S_3_ phase, suggesting that this sample still contained Li. A refinement of the scattering on the tetrahedral sites in the sulfide layers of the OS-II phase was carried out assuming that Cu only was present in the sulfide layers. This produced a composition of Sr_2_MnO_2_Cu_2.2(2)_Li*_x_*S_3_ for this phase OS-II, which is also consistent with the composition obtained in the *in situ* refinements. To account for the remaining Li that had not been removed at this point in the charging process, we calculate that *x* ≈ 1.5(2) in this sample. The sample contained 1.8 moles of crystalline elemental Cu per mole of Mn from the refinement for this *ex situ* sample, broadly consistent with the *in situ* data measured at this point in the electrochemical profile, which was obtained in a separate experiment with a different setup. See Table 1 for similar analysis of the other samples. The uncertainties in the refined and calculated compositions are relatively high considering the multiphase nature of the samples.

During the long 2.5 V process, the lattice parameters of the dominant OS-II phase decrease, but its refined formula weight remains approximately constant. This is consistent with the removal of Li from OS-II, which is not compensated by the re-insertion of Cu, and is expected to lead to the oxidation of Mn. During this process, the amount of Cu present in the oxysulfide phase remains remarkably constant. In turn, the phase fraction of crystalline elemental Cu shows an increase during this process. This observation is ascribed to (i) grain growth of Cu originally extruded as very small particles, (ii) loss of crystallinity of some of the oxysulfide material, and/or (iii) an artifact of the *in situ* experiment. Most importantly, it is clear that the amount of Cu inserted into the oxysulfide does not increase significantly during the 2.5 V process.

At the very end of the 2.5 V process, the *in situ* data show that the phase OS-I is no longer required to model the data satisfactorily. The *ex situ* data obtained for a sample charged at 2.8 V (corresponding to point g in the voltage profile in Figure 2) show that, in addition to the OS-II phase, there is a phase present (OS-III) with a *c* lattice parameter of about 9.8 Å, much smaller than even the smallest value attained in the charge for OS-II of about 11.2 Å (Table 1). This phase could not be reliably identified in the *in situ* data, but in the *ex situ* data, the appearance of this phase, with its intense 001 reflection, is very clear (Figure 5b). The overall composition at point g is refined to be Sr_2_MnO_2_Cu_2.0(2)_Li_0.5(2)_S_3_ and elemental Cu in a 1:1.7(2) ratio.

According to the *ex situ* PXRD and NPD refinements and with the Li content inferred from the position on the charge curve, this OS-III phase constitutes approximately 20 mol % of the oxysulfides present (Figure 5b and Figure 6). Although the refinement of the structure of this phase with its short *c* lattice parameter against the data available is hampered by the fact that it is present only as a minority phase, the contraction of the *c* axis suggest that it has no or few cations present in the sulfide layers and is an oxysulfide with a collapsed sulfide layer, which, in the limit, would have a composition Sr_2_MnO_2_S_3_. Given the small fraction of this phase, it was modeled in the refinements as a collapsed phase with a similar arrangement of ions to OS-I or OS-II, but with the metal ions removed from the sulfide layers, and with shorter S–S distances resulting from the significant shortening of the *c* axis. Attempted refinement of the structure of this minority phase was not possible given the small phase fraction, and further investigations of this phase are needed to determine the coordination environment of the sulfide ions and whether some residual metal ions are accommodated in the collapsed sulfide layer. The extreme composition for OS-III of Sr_2_MnO_2_S_3_ would require that electrons are removed from the sulfide-based valence band and/or sulfide–sulfide bonds are formed. We suggest that this third minority phase (OS-III) is the result of the collapse of the lithiated phase OS-I when all the Li is deintercalated, though further characterization of this phase is required. Given that Li is a feeble scatterer of X-rays and due to the low quality of the neutron data and the fact that sites may be partially occupied by Cu, Li or be vacant, it is not possible to determine using these the diffraction data alone whether any Li remains in the oxysulfide phases (mainly OS-II) after the system has been charged to 2.8 V, but from the position in the charge profile and the NMR results presented below, it is clear that some Li remains in the oxysulfide phases, although it cannot be concluded whether both the majority OS-II phase and the minority OS-III phase contain this remnant Li.

Continued charging to 3.7 V (point i) restores a single oxysulfide phase, and the *ex situ* laboratory X-ray (Figure 5a) and NPD data (Figure 6) show no measurable quantity of crystalline elemental Cu. PXRD and NPD refinements produced a composition of Sr_2_MnO_2_Cu_3.5(2)_S_3_ for this final single oxysulfide phase. The lattice parameters were 0.3% (*a*), 0.15% (*c*), and 0.6% (volume) smaller than for single phase pristine Sr_2_MnO_2_Cu_3.5_S_3_, suggesting that the product may be slightly Cu-deficient relative to the original starting material.

#### 3.2.3 Second Discharge

When samples were discharged to 1.1 V, charged to 2.8 V, and then discharged, the phases formed were found to be similar to those produced during the first charge processes (Figure 5e,f). Such similarities are consistent with the observation that the voltage trace during this second discharge mirrors that of the first charge (Figure 2a). Furthermore, the sample obtained after the 2.2 V process on discharge corresponded closely to that obtained at the same state on charging, as shown side by side on Figure 5c and Figure 5e, respectively. However, at point *m*, the structural refinement was found to require two phases, one of which was Cu-rich (Figure 5f). Therefore, the second discharge is not as efficient a process of Cu extrusion as the first. We suggest that the structural changes required to precipitate metallic particles out of a framework introduces a strain that damages the particles and renders them less active upon subsequent cycling. Upon cycling, this electrochemical grinding, coupled with copper dissolution, is expected to continue, contributing to the capacity loss reported in a previous report.^22^

### 3.3 *Ex Situ* XANES Analysis

#### 3.3.1 First Discharge

**Figure 7:**
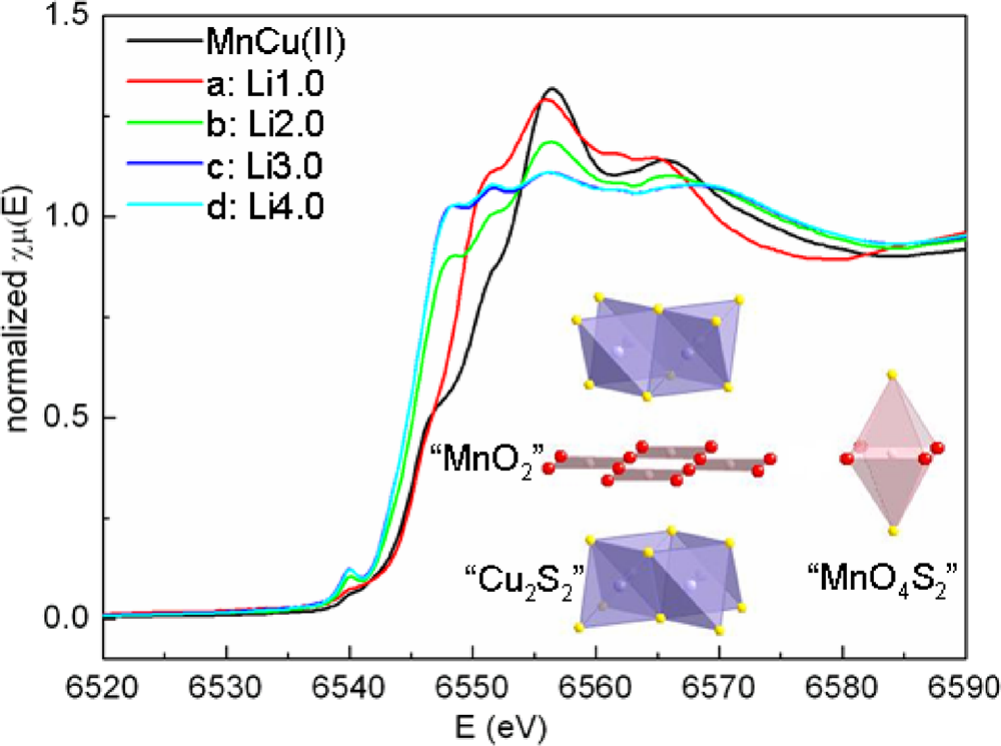
Normalized Mn K-edge XANES spectra of pristine and lithiated MnCu(II) phases during the first discharge. The inset shows the distorted octahedral environment around Mn (represented as “MnO_4_S_2_” in the figure) (Sr ions are omitted for clarity).

The mean Mn oxidation state in MnCu(II) is +2.5 based on the size of the effective moment per Mn ion in the Curie–Weiss region and XANES measurements.^20^ On electrochemical Li insertion, the Mn K-edge absorption shifts to a lower energy (Figure 7), indicating that Mn is reduced during discharge. This process of reduction is largely complete at sample b (Li2.0), halfway through the first discharge. Thereafter, the absorption edge does not show a significant shift but a noticeable change in shape that is ascribed to structural changes in the local coordination of Mn. In MnCu(II), Mn is located in an axially distended octahedral site with four oxide and two sulfide ions in the equatorial and axial positions, respectively. As Li is inserted into the sulfide layer and Cu is extruded from it, significant changes to the sulfide environments are expected, which in turn may influence the Mn environment. The normalized Cu K-edge XANES spectra of MnCu(II) during the first discharge and their corresponding first derivatives (Figure S5) show a slight broadening but very little shift in the Cu K-edge when the sample has been discharged to point b (Li2.0). According to the *in situ* PXRD measurements, less than half the Cu has been extruded as the element at that point, so the Cu edge spectrum will have contributions from elemental Cu and Cu^+^ in the oxysulfide phase, consistent with the broadening of the first derivative of the XANES spectrum at point b. Beyond 2 Li, the Cu edge more noticeably shifts to a lower energy (Figure S5) until the end of the discharge (point d) as Cu^+^ is reduced and the metal is completely extruded.

These XANES results and the *in situ* PXRD results rule out a model in which all the vacant tetrahedral sites in the sulfide layers are filled prior to any Cu extrusion. The insertion of 0.5 moles of Li to fill all these sites would be sufficient to reduce Mn all the way to the +2 state. However, Mn reduction persists at least up to the point at which 2 moles of Li per Mn ion have been inserted. The initial process at 2.2 V (Figure 2b) corresponding to approximately 0.1 Li is nonetheless likely to be associated with Li insertion only into the vacancies (as discussed below).

**Figure 8:**
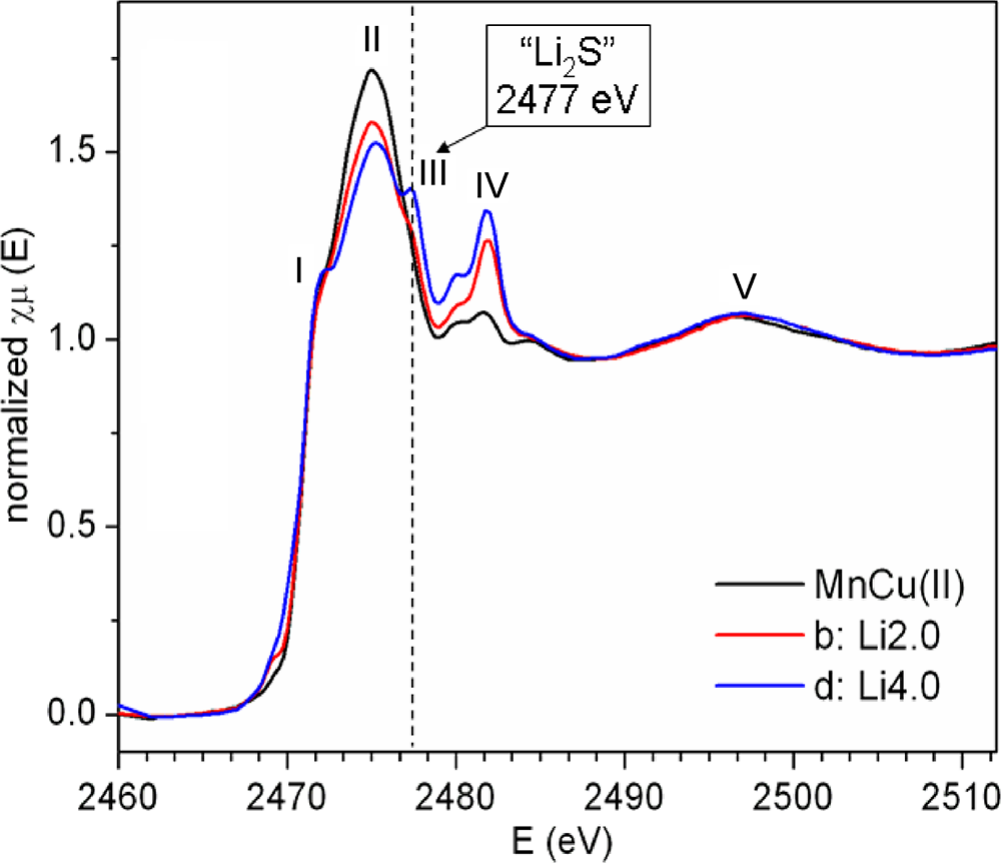
Normalized S K-edge XANES spectra of pristine and lithiated MnCu(II) phases during the first discharge. The absorption features are labeled with numbers I–V. The energy of feature III (ascribed to sulfur in a Li_2_S-like, anti-fluorite arrangement) is indicated with a dashed line.

Two different environments exist for S in the structure. One-third of the S atoms lie in the center of the sulfide layers directly coordinated only to Cu (and/or Li). The other two-thirds of the S atoms are coordinated to both Cu/Li and Mn. The XANES spectra at the S K-edge (Figure 8) contain contributions from both species. The lowest energy shoulder I at 2471 eV is assigned to transitions of the S 1s electrons to transition metal-3d/S-3p states, whereas the features at higher energies (II–IV) are associated with mixed states involving metal 4s and 4p.^36,37^ Upon lithiation, the intensity of the peak II absorption (at 2475 eV) decreases while that of IV (2482 eV) increases. Similar changes in intensity have been observed earlier for Li*_x_*TiS_2_.^36^ Computational simulations have concluded that these changes could be primarily by an elongation of the Ti–S bond during reduction, together with a decrease of the empty states available for excitation of S 1s electrons due to the introduction of electrons during the reduction process. A 5.5% elongation of the Mn–S bond resulting from the chemical lithiation of MnCu(II) has earlier been reported. In the present study, insertion of Li into MnCu(II) produces a change in the chemical bonding between S and its surrounding cations; however, the S K edge XANES data are consistent with the absence of major crystal structural reorganizations, with most changes occurring in the electronic structure, especially near the Fermi level.

In addition to these changes, a new peak at 2477 eV (III) emerges during discharge, which resembles the signals reported for anti-fluorite-type Li_2_S,^34,36^ consistent with the formation of a Li_4_S_3_ anti-fluorite-type slab. Given their similar ionic radii,^35^ the substitution of Cu^+^ by Li^+^ is not expected to introduce substantial structural changes, but the ionic character of the bonding within the antifluorite slabs will change.^36^

#### 3.3.2 First Charge

**Figure 9:**
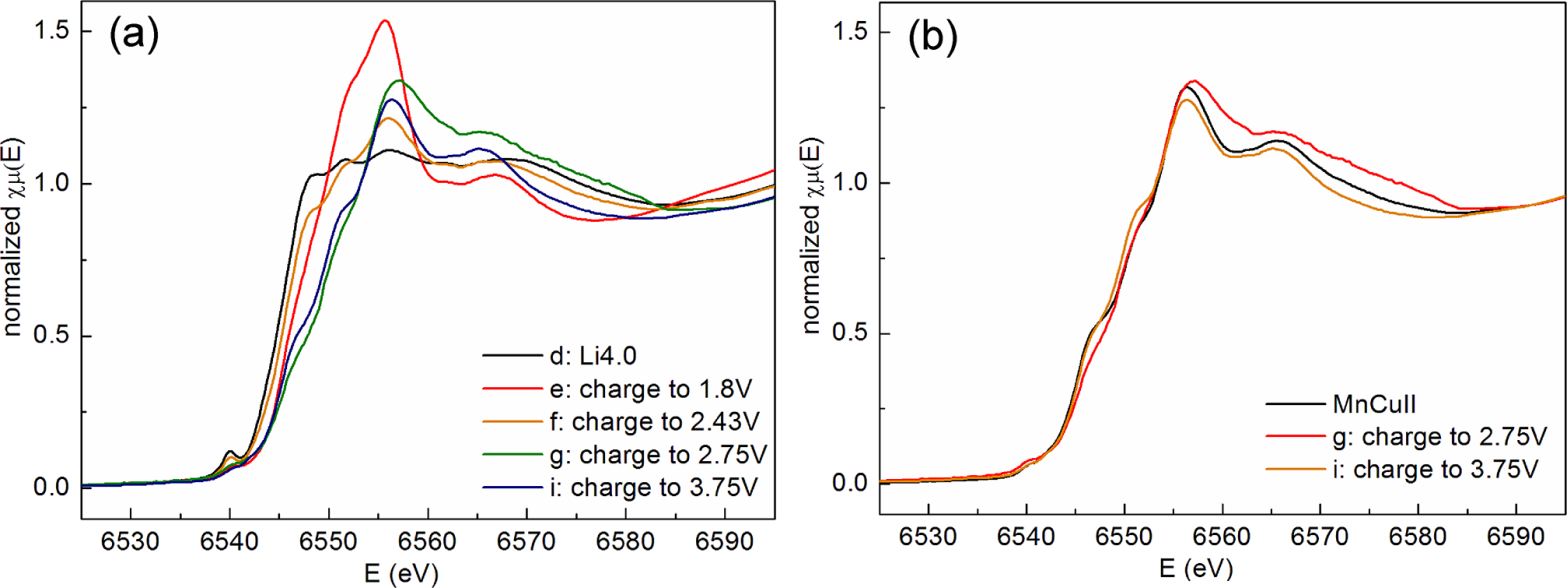
Normalized Mn K-edge XANES spectra of (a) lithiated MnCu(II) phases during the first charge and (b) MnCu(II) along with samples at points g and i.

The normalized Mn K-edge XANES spectra of different states of charge of MnCu(II) are shown in Figure 9. On charging from the fully lithiated sample (point d in the voltage profile in Figure 2) to the end of the 1.8 V process (point e), the absorption edge clearly shifts to higher energy (associated with a significant change of the line shape), indicating the oxidation of Mn during the 1.8 V process upon Li removal, which is not fully compensated by the intercalation of Cu. Charging further to the start of the 2.5 V process (point f), the absorption edge of Mn slightly shifts back to lower energy. This re-reduction of Mn between point e and f coincides with the *in situ* PXRD observations of a sharp increase in the overall content of the Cu-rich OS-II phase at the expense of OS-I and a fairly rapid decrease in the weight fraction of elemental Cu in the sample. However, from the fully lithiated sample d to sample f, there is only a small net shift of the Mn absorption edge. On further charging, there is a large shift in the position of the Mn edge to higher energies during the 2.5 V process (point f to point g).

The PXRD and NPD results show that there is little change in the amount of elemental Cu in this region and Li deintercalation is not accompanied by Cu^+^ ion insertion into the oxysulfide. Correspondingly, the XANES results show that Li deintercalation during the 2.5 V charging plateau is accompanied by Mn oxidation, also consistent with the decrease in unit cell volume in the diffraction data. On charging up to 3.75 V (point i), the final spectrum is nearly similar to that of MnCu(II) (Figure 9b), indicating that de-lithiation from the structure and the re-insertion of Cu largely restores both the oxidation state and the environment surrounding Mn, consistent with the diffraction results.

No significant change in Cu K-edge position on charging from the fully discharged state (sample d) up to the onset of the 2.5 V process (sample f) was observed (Figure S6). However, charging to 2.75 V (sample g) and 3.75 V (sample i) induces a continuous but small shift of the Cu K-edge to a higher energy to a position close to that of MnCu(II). This final insertion of Cu into the oxysulfide occurs at a significant overpotential (see Figure S1) and also at a higher oxidation potential than standard Cu/Cu^+^ oxidation vs Li/Li^+^ (3.56 V).

Figure S7 shows the S K-edge XANES spectra for samples obtained during the first charge. Feature III, characteristic of sulfide in an antifluorite Li_2_S-like environment, decreases in intensity (Figure S7a, inset) during both the 1.8 and 2.5 V processes and is not visible at 2.75 V (sample g), suggesting that a large amount of Li has been removed. The process at 2.5 V (*i.e*., sample f to sample g) involves an increase in the intensity of feature II, presumably arising from the shortening of the Mn–S bonds resulting from the oxidation of Mn. The absorption spectra for samples g and i are similar to each other, but they exhibit clear differences from the spectrum of pristine MnCu(II) (Figure S7b), suggesting that the Cu–S bonding as well as the sulfur environments are not entirely restored to that of the pristine material even when the battery was charged to 3.75 V. Besides the intensity differences of features II and IV, a new absorption at around 2470 eV is present for both samples g and i (inset in Figure S7b). The appearance of a peak at a similar energy has earlier been ascribed to the partial oxidation of S^2–^ to S^2–*n*^ (0 < *n* < 1) for NaCrS_2_^38^ and Li_2_FeS_2_.^39^ This sulfur oxidation (likely coupled with Mn oxidation) may help stabilize the cation-deficient OS-III phase formed during the 2.5 V charging process.

#### 3.3.3 Second Discharge

**Figure 10:**
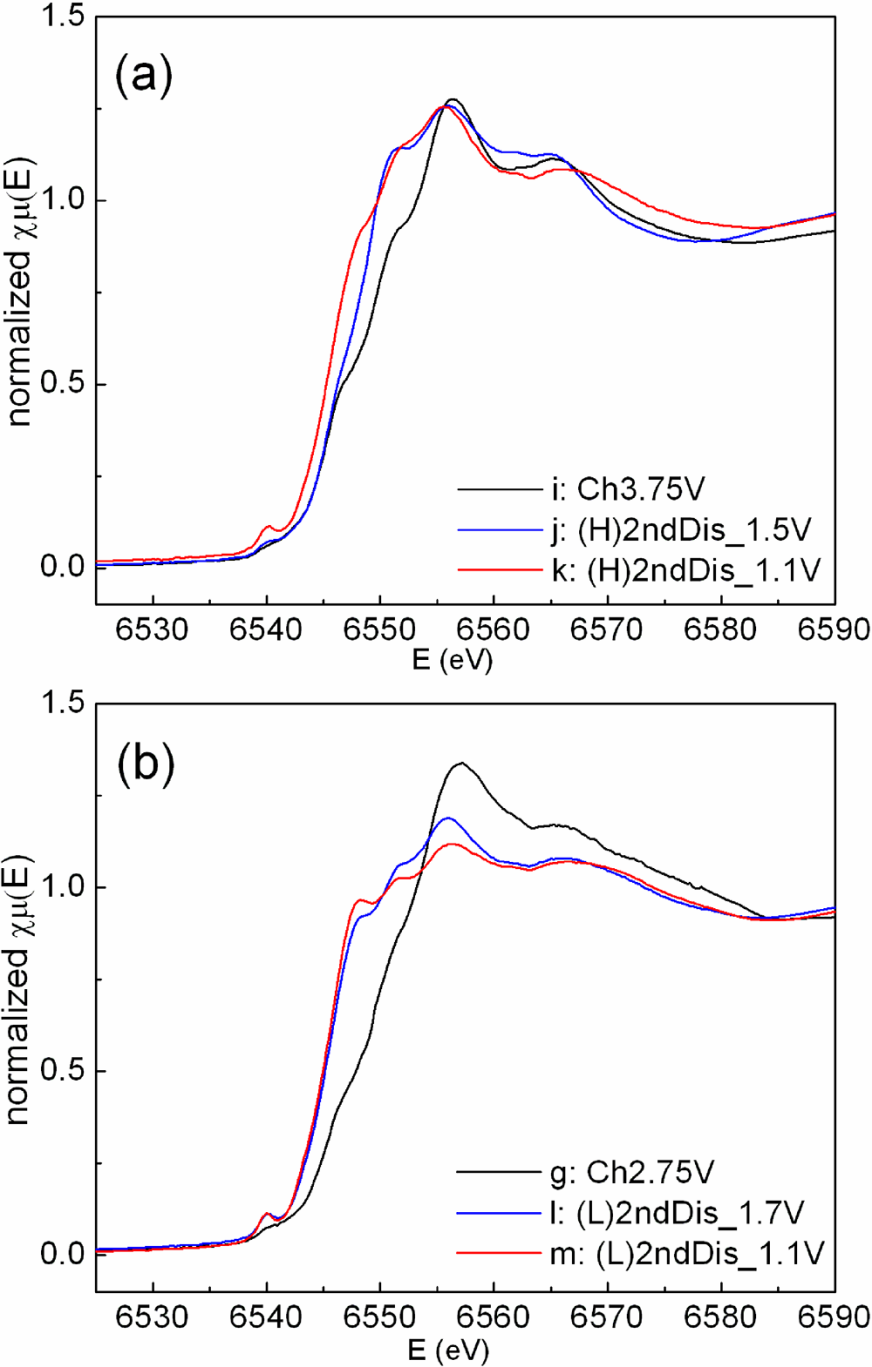
Normalized Mn K-edge XANES spectra of MnCu(II) phases during the two second discharge processes after the first charge. The cutoff voltage of the first charge is (a) 3.75 V and (b) 2.75 V.

The voltage profile during the second discharge varies depending on the cutoff voltage applied for the first charge (2.75 or 3.75 V). The Mn K-edge XANES spectra obtained during the second discharge after charging to 3.75 V (Figure 10a) resemble those obtained during the first discharge of the pristine oxysulfide with Mn fully reduced, as could be expected from the similar electrochemical signatures observed during both cycles. When the cutoff voltage applied to the first charge is 2.75 V, the Mn XANES results clearly show that the absorption edge moves to lower energy only from sample g to l but stays at the same position thereafter (Figure 10b). This suggests that Mn reduction takes place mostly during the 2.2 V process (sample g).

The Cu K-edge XANES data shows when the battery is charged to 3.75 V, the behavior on discharge is similar to that of the first discharge *i.e.*, Cu reduction is not evident when discharging the battery to 1.5 V (*ca.* 1.0 Li inserted, sample j, Figure S8). When the first charge is to 2.75 V, distinct plateaus are found at 2.2 and 1.5 V on the subsequent discharge, with continuous Cu reduction observed throughout (Figure S8).

Figure S9 compares the S K-edge XANES spectra during the second discharge for materials charged to either 3.75 or 2.75 V. While similar features are observed in the two cases, comparison of points obtained during the discharges shows that the S atoms undergo greater changes during the second discharge after being charged to 3.75 V than after a 2.75 V charge.

### 3.4 ^7^Li NMR Results

**Figure 11:**
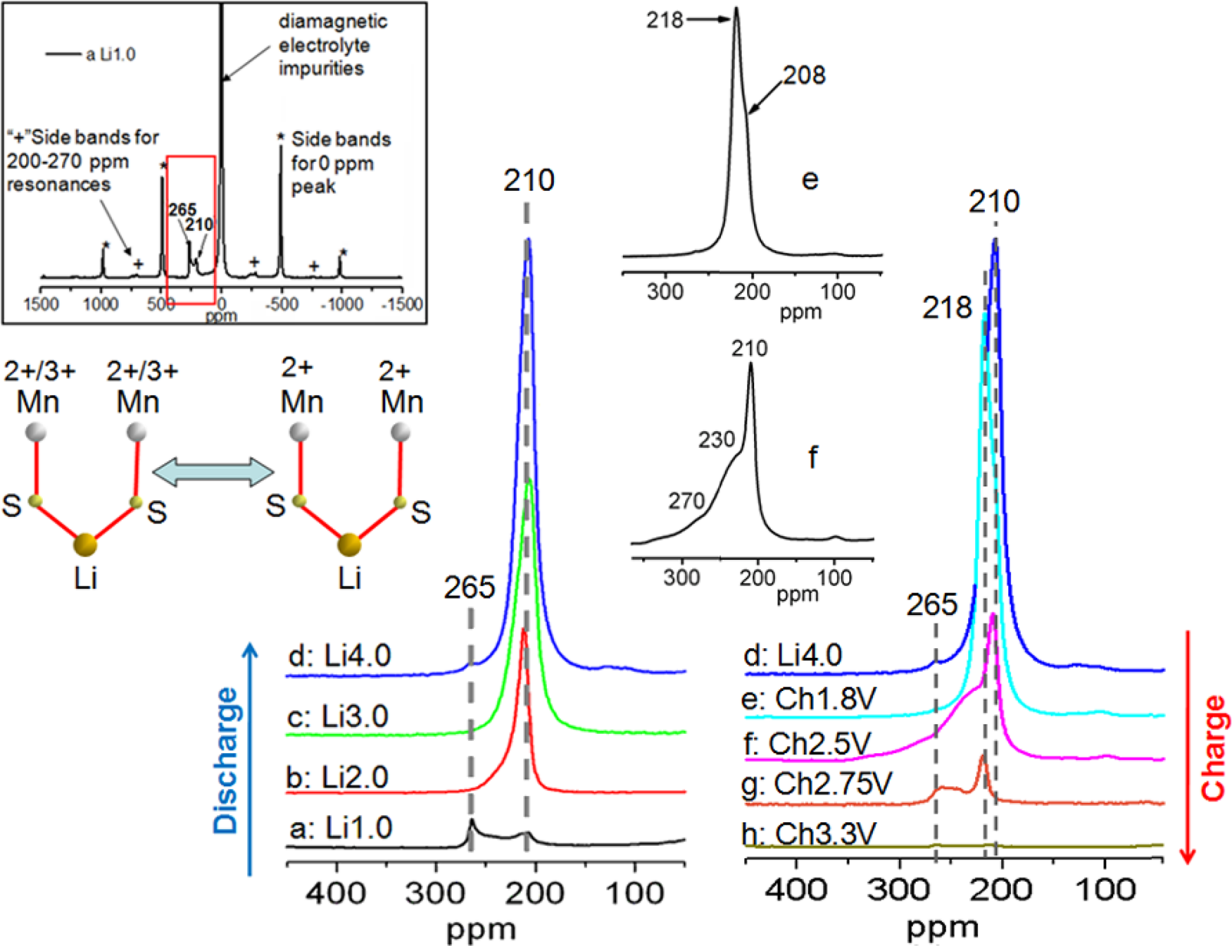
Zoom-in view of the ^7^Li NMR spectra of MnCu(II) during the first discharge (left) and charge (right). The spectra have been normalized based on the acquisition number and sample mass. The inset on the top left corner is the full spectrum of sample a: Li1.0 shown as an example. The zoomed region is indicated by a red rectangular box. The spectra of samples e and f within the 50–350 ppm region are shown separately in the middle inset. A schematic picture showing the Li environments in the MnCu(II) structure during the discharge/charge cycle is also included (left lower part).

The *ex situ*^7^Li NMR spectra for different samples recovered during the first discharge and charge are shown Figure 11. Besides an intense resonance at 0 ppm, which arises from diamagnetic impurities/electrolyte decomposition products, all the spectra show a group of resonances between 200 and 270 ppm, the shift resulting from the Fermi contact interaction between paramagnetic Mn and Li ions, mediated through the S bonds via a similar mechanism to that seen in lithium manganese oxides.^40^

During the first discharge, the spectrum of sample Li1.0 (sample a in Figure 2b) shows two distinct resonances at 210 and 265 ppm, and upon further Li intercalation, only the former remains in the spectra of samples Li2.0 (b), Li3.0 (c), and Li4.0 (d). The intensity of the 210 ppm resonance increases with Li content. Li is located in the tetrahedral sites within the antifluorite-type layer and interacts with two Mn ions in the oxide layer via Mn–S–Li interactions. Previous Mn K-edge XANES, magnetic susceptibility and bond valence sum data revealed a mean oxidation state of Mn^2.5+^ in the pristine phase.^20^ Mn ions are coupled ferromagnetically within the MnO_2_ planes and magnetic long-range order occurs below about 50 K. This ferromagnetism, which arises either from superexchange between charge-ordered Mn^2+^ and Mn^3+^ ions or from itinerant d_*x*^2^ – *y*^2^_ electrons, is further evidence for the mixed valence of Mn. As Li is inserted into the framework, the XANES results indicate that full reduction to Mn^2+^ is obtained in sample Li2.0 (b). Therefore, the 210 ppm resonance has been assigned to Li interacting with only Mn^2+^ (two Li–S–Mn^2+^ interactions) and the 265 ppm resonance has been assigned to a Li environment containing Mn^3+^ (one Li–S–Mn^3+^ interaction and one Li–S–Mn^2+^ interaction). An environment with Li interacting with two Mn^3+^ ions is unlikely. First, this configuration is not energetically favorable due to the larger Coulombic repulsion between two more highly charged Mn^3+^ cations. Second, the amount of Mn^3+^ is very small after insertion of 1 mol Li. Since the NMR shifts are additive, it can be deduced that each Li–S–Mn^2+^ interaction results in approximately a 210 / 2 = 105 ppm shift in the ^7^Li NMR spectrum and each Li–S–Mn^3+^ induces around a 265–105 = 160 ppm shift. The value for the Li–S–Mn^2+^ interaction is consistent with the shifts observed for the other lithiated oxysulfides in the same series as MnCu(II).^22^ The Li Fermi contact shifts in paramagnetic materials are usually proportional to the unpaired electron density transferred from the paramagnetic species. In the axially elongated MnO_4_S_2_ octahedra the electronic configurations are as follows: Mn^2+^: (d*_xz_*)^1^,(d*_yz_*)^1^(d*_xy_*)^1^(d_*z*^2^_)^1^(d_*x*^2^ – *y*^2^_)^1^ and Mn^3+^: (d*_xz_*)^1^,(d*_yz_*)^1^(d*_xy_*)^1^(d_*z*^2^_)^1^(d_*x*^2^ – *y*^2^_)^0^. The electron counts only differ in the d_*x*^2^ – *y*^2^_ orbitals, which cannot participate in the Mn–S–Li interactions. Therefore, the larger shift for contacts with Mn^3+^ seems to simply be a consequence of the greater covalency of the Mn–S interaction than in the Mn^2+^ case.

The signal at 210 ppm comes from the OS-I phase, the only Li-containing oxysulfide present in Li4.0 (d). During the charge, from sample d to e, an intense resonance appears at around 218 ppm (Figure 11), the shift likely arising from the increase in the oxidation state of Mn in the structure. Upon further charging to 2.5 V (f), the 210 ppm signal recovers, indicating the restoration of some Mn^2+^ environments nearby to Li, and broad shoulders at higher chemical shifts (centered at 230 and 270 ppm) appear. These are consistent with the Mn XANES results (see Figure 9a), which indicate oxidation of Mn during initial charging to 1.8 V and then partial reduction of Mn as the charging is continued to 2.50 V (f). *In situ* PXRD results show the OS-II phase appears during 1.8 V charging, accounting for the appearance of 218 ppm resonance at point e. When the battery is charged further to 2.75 V (sample g), the spectrum shows a low intensity resonance at 218 ppm and a further broad resonance feature centered around 250 ppm, consistent with the oxidation of Mn as clearly observed in Mn XANES. The 210 ppm resonance is absent at point g, congruous with the diffraction findings that no more OS-I is present at this point.

The higher ppm resonances are assigned to Li in environments containing both Mn^3+^ and Mn^2+^. The broad lines evident on charging from point e to point g suggest that Li is distributed over environments where a continuous range of bond lengths (Mn–S and S–Li), bond angles (Li–S–Mn), and Mn oxidation states are present, resulting in a continuous distribution of the shifts. The diffraction results show that two phases are present in this region, and although the precise distribution of Li between these phases cannot be quantified by analysis of our diffraction measurements, it is likely that both OS-I and OS-II phases contain some Li over much of this 2.5 V process, with the third phase observed at the very top of charge not containing any Li. Clearly, the presence of Li in at least two phases contributes to there being a wide range of environments.

**Figure 12:**
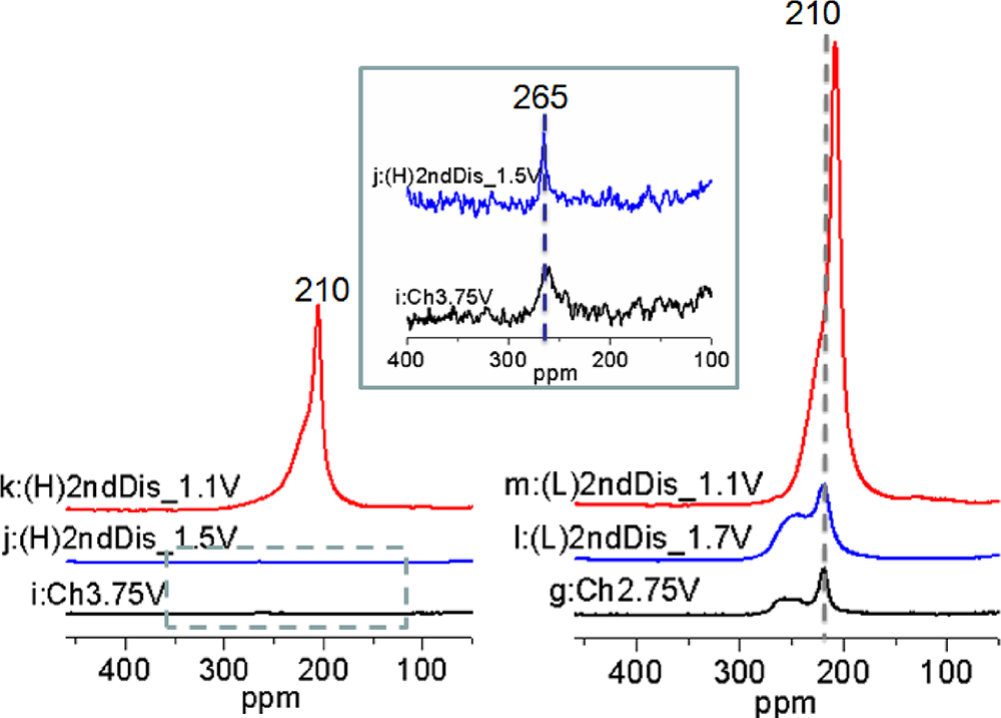
^7^Li NMR spectra of MnCu(II) during the second discharge after charging to 3.75 V (left) and 2.75 V (right). The spectra have been normalized based on the acquisition number and sample mass, and the two groups of spectra are shown at the same scale. The inset shows an expansion of the spectra of samples i and j (indicated by a gray rectangle box in the left) 20 times amplified in magnitude.

The resonances in the spectra of the samples charged to 3.3 V (sample h) and 3.75 V (see Figure 12, sample i) have only a very weak resonance at 265 ppm, indicating that only a small amount of Li remains in the structure at this stage and that the Mn +2.5 state has been restored. Consistent with this, the S XANES results show that the environment resembling that in Li_2_S is not observed in samples charged to higher than 2.75 V, and the NPD result on sample i shows that a composition close to Sr_2_MnO_2_Cu_3.5_S_3_ is restored.

The ^7^Li NMR results for samples during the two second discharge processes are shown in Figure 12 (N.B.: the small displacement of the shift in the spectrum for sample m is not intrinsic but very likely caused by the small temperature variations between the MAS experiment). It is clear that the spectral intensity of the samples following a charge to 3.75 V is significantly lower than those where the cutoff voltage was set to 2.75 V. This is consistent with the electrochemical profiles shown in Figure 2, where the Li equivalents in samples j and k are smaller than in samples l and m. Despite the intensity difference, the spectra of the two samples at the end of each discharge (samples k and m) have very similar line shapes: an intense resonance at around 210 ppm with a shoulder at a higher frequency. This observation contrasts with the symmetric resonance at 210 ppm observed for the sample at the end of the first discharge (sample d: Li4.0). It further confirms that the reduction of the material does not appear to be as efficient after the first cycle, as discussed above in the context of the PXRD data (Table 1).

## 4 Discussion

**Figure 13:**
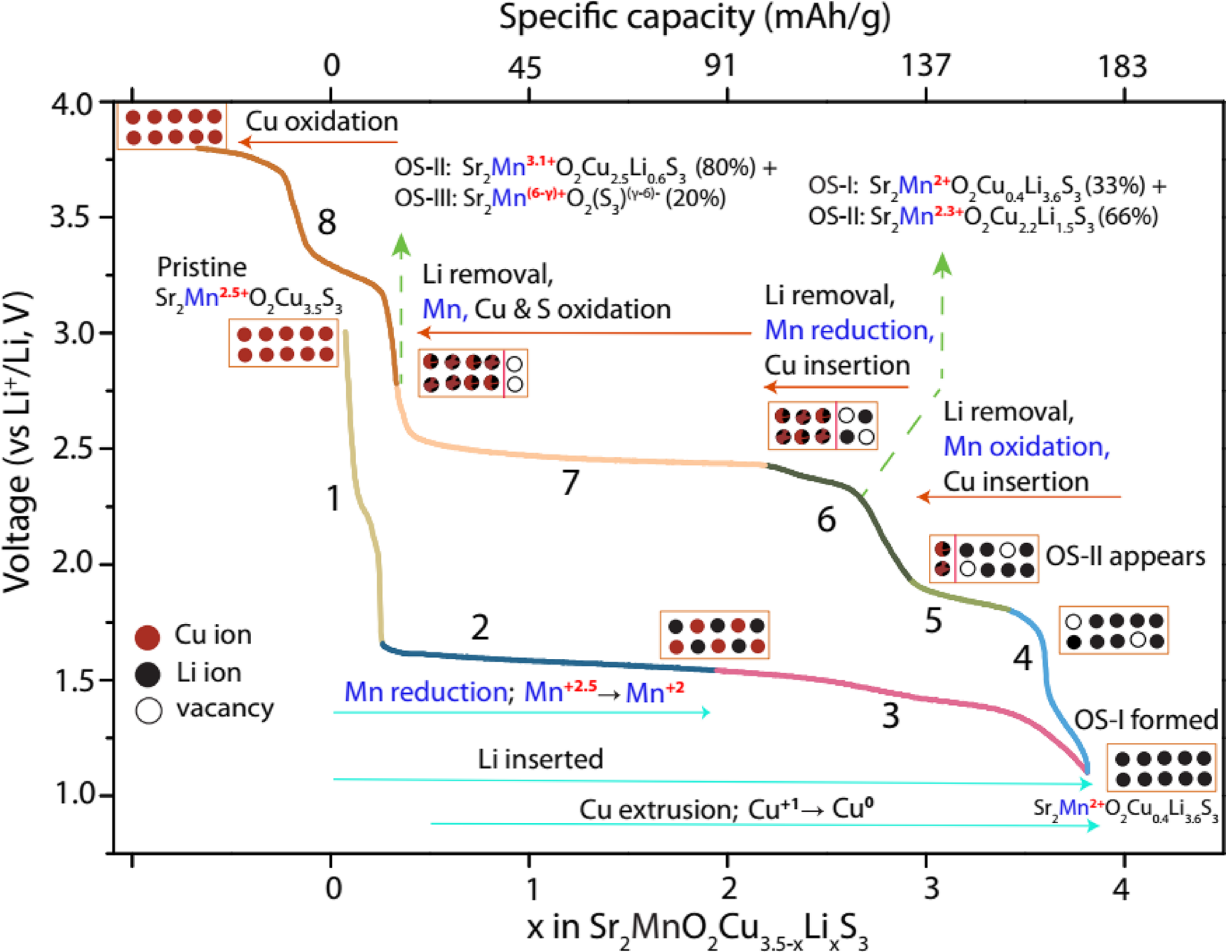
Summary of the redox reactions involved for each process during the first cycle of a MnCu(II)|Li battery within the voltage window of 1.1–3.75 V. The discharge and charge processes are labeled with cyan and red arrows, respectively. The arrow indicates the direction of voltage change. The redox changes associated to the particular voltage–capacity regime are given over each arrow. The voltage–capacity plot is divided in eight steps, each marked with different colors, to help visualize the different redox processes. The orange boxes represent model of the Li/Cu content in the oxysulfide phases, where Cu^+^, Li^+^, and vacancies are shown by red, black, and unfilled balls, respectively. Mixed Cu and Li occupancy is illustrated by fused red/black balls. Where a red line inside the box is shown, this indicates the coexistence of two phases. This model is a simplistic representation of the insertion/extraction of Cu/Li ions and formation of vacancies at different redox potentials. The variable γ reflects uncertainties in the oxidation state of Mn and S of the phase OS-III, which arises as OS-III is a minority, and hence a less well-characterized phase, the composition Sr_2_MnO_2_S_3_ representing the limiting one, assuming all metal ions are indeed removed from the sulfide layer.

The redox reactions and phase transitions occurring during the first discharge and charge cycle of a Li battery containing MnCu(II), as deduced from our results, are summarized in Figure 13. The pristine MnCu(II) structure is composed of rigid perovskite-type oxide (Sr_2_MnO_2_) layers and relatively flexible antifluorite-type sulfide layers (Cu_4−δ_S_3_). The thermodynamically stable composition obtained in conventional high temperature solid-state synthesis has Mn in an oxidation state of +2.5, and there are a corresponding number of Cu vacancies in the sulfide layer (δ ≈ 0.5). The oxide and sulfide layers are commensurate with one another so the S–S separation within the basal plane of the sulfide layer, and the Mn–Mn distance within the oxide layer are both equal to the basal lattice parameter, *a* of 4.015 Å (which is also double the Mn–O bond length (2.0076 Å)).^22^ This S–S distance is slightly longer than the S–S distance in antifluorite Cu_2_S of 3.94 Å,^41^ so the tetrahedral sites are larger in MnCu(II) and the Cu ions are highly disordered over the tetrahedral sites and neighboring trigonal sites, likely associated with considerable mobility of Cu.

When Li is inserted into the structure, on first discharging the cell, there is a short process at 2.2 V in the electrochemical profile (step 1, Figure 13), ascribed to the insertion of 0.1 Li per Mn into the vacant sites in the sulfide layer (*i.e.*, sufficient to fill only 20% of the vacant tetrahedral sites). During the following long gently sloping (approximately) 1.7 V process, Mn is reduced to Mn^2+^ as Li fills the remaining vacancies and also displaces elemental copper; the XANES and *in situ* PXRD results show that these processes occur concurrently up until about halfway through the discharge (step 2) at which point the XANES results show that Mn is fully reduced. The PXRD results show that copper extrusion continues until the system is fully discharged. During this 1.7 V process a single oxysulfide phase, OS-I is observed, indicating a continuous change of the parameters of the framework as lithium is inserted and copper is extruded. At the end of the discharge, the basal lattice parameter increases sharply (Figure 4a) as the OS-I phase becomes fully lithiated Sr_2_MnO_2_Li_4_S_3_ with no vacancies on the tetrahedral sites in the sulfide layer (Table S2).

During the charge, the mechanism of delithiation and eventual reinsertion of Cu is complex. During the first process at *ca.* 1.8 V, only Li is removed, indicated by a decrease in the intensity of the S XANES peak corresponding to Li_2_S-like sulfide ions and the diminishing intensity of peaks in the ^7^Li MAS NMR spectra. However, the PXRD results suggest that re-insertion (oxidation) of Cu during this process is negligible, which is ascribed, at least in part, to the sluggish migration of Cu^+^ into and through the structure to occupy the tetrahedral sites vacated by Li^+^, the Li^+^ ions being more readily removed from the structure. The shift in the Mn K-edge and the shift of the ^7^Li NMR resonance indicates that the charge compensation during the 1.8 V charging process comes from the oxidation of Mn. Importantly, during the 1.8 V process, PXRD shows the clear presence of two oxysulfide phases (end of step 5). The “step” in the charging profile between 1.8 and 2.5 V (point e to point f) is accompanied by a rapid change in the phase fractions of the two oxysulfide phases and by a decrease in the amount of elemental Cu present in the diffractograms (step 6). A slight re-reduction of Mn, as the Cu is inserted into the OS-II and OS-I phases, is revealed by the behavior of the Mn K edge and the ^7^Li NMR resonance. The GITT data (Figure S1) indicates a large overpotential for step 6, the voltage relaxing back to close to that of step 5. It is possible that some of the Cu insertion occurs via a chemical process during stage 6, resulting in the Mn reduction.

The process at 2.5 V involves the continued removal of Li, but the NMR results show that there still exists a small amount in the structure even at 2.75 V. During this process, the Mn K edge undergoes a large shift to higher energies, the oxidation of Mn resulting in a short Mn–O bond length as revealed by the short *a* lattice parameter for the majority oxysulfide phase. There is very little change in the amount of Cu in the crystalline oxysulfide phases. The unit cell volume of the majority oxysulfide phase, OS-II, reaches a minimum toward the end of this process consistent with the formation of a new derivative of Sr_2_MnO_2_Cu_3.5_S_3_ with a larger number of vacancies on the tetrahedral sites in the sulfide layers and with a higher Mn oxidation state. As the battery is charged to 2.75 V, there is evidence in the *ex situ* PXRD data for a further related oxysulfide phase (OS-III), which has a greatly contracted (by 14%) *c* lattice parameter compared with the Sr_2_MnO_2_Cu_3.5_S_3_ parent phase (end of step 7). This was only ever found as a minority phase, hampering the detailed analysis of the structure, but the large contraction of the lattice parameter normal to the layers suggests that it may be completely devoid of metal cations in the sulfide layer. Further experiments to isolate as bulk phases this phase and the other intermediate phases evident in the experiment are ongoing.

The deintercalation of large amounts of Li over the course of the long 2.5 V process does not appear to be compensated completely by the re-injection of Cu, and thus oxidation of Mn alone is not sufficient to compensate the oxidative deintercalation of Li (step 7). The PXRD and NPD results at 2.75 V (point g) confirm that the insertion of Cu is far from complete and the majority of the Cu exists in the elemental form rather than being reinserted into the oxysulfide phase, and more vacant tetrahedral sites must exist in the oxysulfide phase OS-II than are present in the pristine phase (Table S2). It is clear that Li removal occurs faster than Cu reinsertion. The Cu XANES K-edge at point g suggests that the Cu is in the more oxidized state than in the pristine material. The S K edge XANES also develops new edge features, which have been ascribed to the partial oxidation of S^2–^ at the end of 2.5 V charging. The minority phase OS-III with the contracted *c* parameter, consistent with a lack of cations in the sulfide layers, would have a limiting composition Sr_2_MnO_2_S_3_, a composition that cannot be fully accounted for by assuming Mn oxidation, and at least partial sulfide oxidation in this phase is consistent with the XANES data. The majority phase at this point in the charge process, OS-II has an estimated composition Sr_2_MnO_2_Cu_2.5(2)_Li_0.6(2)_S_3_, based on its position in the charge profile and because the NMR results suggest that some Li ions remains at this composition. This is the limiting composition assuming that no Li or Cu ions are present in the minority phase OS-III, so the OS-II phase also has at least one quarter of the tetrahedral metal sites in the sulfide layer vacant and is highly oxidized. Thus, we suggest that charge balance is achieved by residual Li, and partial oxidation of Cu (above 1+), S (above 2−), and Mn (given the number of variables, only the Mn oxidation state is used in Figure 13 for charge balance, *i.e.*, the Cu is assumed to be all Cu^1+^ and thus Mn has an average oxidation state of 3.1). It is likely that the Li removal and only partial reinsertion of Cu, resulting in a phase with an oxidation state of Cu beyond 1+ and/or Mn above 2.5+, result in the higher voltage of step 7 (2.3 V from the GITT, Figure S1), in comparison to that of step 5 (1.7 V). The transition from the voltage associated with step 5 (which is also similar to that seen in the first discharge) to that of step 6 and then step 7 occurs when Mn has been oxidized to close to Mn^2.5+^ and Cu reinsertion cannot keep track with Li removal.

Discharging from the manganese oxysulfide OS-II and OS-III phases that are present at the end of the 2.3 V process purely involves reductive Li insertion into vacant sites in the sulfide layer (presumably in both the highly Cu-deficient oxysulfide OS-II and the “collapsed” oxysulfide phase OS-III). This results in more capacity than seen for (2.2 V) step 1 in the discharge of the pristine phase, consistent with Li filling this large number of vacant sites with an accompanying reduction of Mn (and Cu). Furthermore, the sample obtained at the end of the 2.2 V process during the second discharge indeed contains a similar amount of Cu to its precursor charged to 2.75 V (g) (see Table 1), suggesting that extrusion of Cu is low until the lower voltage process commences. Hence, in the model proposed here, when the system is charged up to 2.75 V, the 2.2 V process on discharge corresponds to pure Li intercalation/deintercalation into oxysulfide phases.

Upon charging from 2.75 to 3.75 V (step 8), the remaining Li is removed and further oxidation of largely all the Cu is clearly evident from the absence of crystalline elemental Cu upon charging to 3.75 V observed in the PXRD and NPD measurements although the Cu and S K-edge XANES measurements suggest that there are small differences between the pristine Sr_2_MnO_2_Cu_3.5_S_3_ and the material obtained by charging up to 3.75 V. It is clear that a notable amount of copper is only reinserted into the oxysulfide at these higher potentials. The overpotential required for reinsertion is ascribed to the high activation energy of Cu insertion (and lower mobility of the Cu^+^ ion) than Li^+^ migration so that the Mn ions are oxidized to their maximum stable oxidation state before Cu insertion. This has analogies with many conversion chemistries, where the sluggish TM (vs Li) migration results in a hysteresis between charge and discharge.^42,43^ A more closely related, so-called “path hysteresis” mechanism is seen for the Ti spinel CuTi_2_S_4,_ which undergoes a CDI reaction to form LiTi_2_S_4_ + Cu on discharging. The charge reaction then occurs at a higher voltage than the discharge voltage, the voltage being set by a different reaction, namely, the Li removal reaction to form Ti_2_S_4_.^42^ The different reactions on discharge and charge have been ascribed to the much lower mobility of the Cu^1+^ ions than Li^+^ ions in the spinel structures.^12,42^ Cu reinsertion in this system is partially accomplished via a chemical reaction.^15^

Overpotentials that will also induce Cu dissolution into the electrolyte are required to complete the reaction in the Sr_2_MnO_2_Cu_3.5_S_3_ system. Although the diffraction data suggest that the composition Sr_2_MnO_2_Cu_3.5_S_3_ is largely restored, the uncertainty in the refined composition (∼0.2 Cu) does not rule out that some loss of Cu occurs due to this process of dissolution into the electrolyte during reinjection.

Upon subsequent discharge from this highly charged state, the electrochemical response mirrors that of the first discharge, contrary to what happens upon reduction from 2.75 V. It is interesting to note that the capacity at 2.2 V at the start of the discharge process is twice as much in the second discharge as in the first, further supporting the existence of more Cu vacancies in the overcharged sample than in the pristine material and thus the possibility of Cu loss from the electrode material due to dissolution.

These results clearly indicate that the amount of Cu that needs to diffuse in and out of the active material particles is noticeably smaller when the cutoff voltage on charge is set at 2.75 V. It is possible that cycling over smaller variations in Cu content will result in the introduction of less mechanical strain into the electrode, thereby providing a good explanation for the rather better cycling performance of MnCu(II) over the small voltage window than when cycled up to 3.75 V. An additional source of capacity loss in the latter conditions relates to the dissolution of Cu ions. The evidence presented here strongly suggests that some of these ions are never reinjected into an oxysulfide. Thus, they can easily diffuse through the electrolyte over to the anode side, where they will be reduced by the very low potentials at which it operates. The result would be the plating of Cu onto the Li metal electrode used in the experiments and redox shuttle mechanisms.

## 5 Conclusions

Layered oxysulfides Sr_2_MnO_2_Cu_2*m*–δ_S_*m*+1_ (*m* = 1, 2, and 3, δ ≈ 0.5) with alternating perovskite-type [Sr_2_MnO_2_] layers and antifluorite-type [Cu_2_S] layers in the structure show reversible reactivity toward Li via a displacement mechanism. Measurements on the *m* = 2 member made both during the electrochemical cycling and after stopping the cycling at various points reveal that the discharge proceeds both with Mn reduction in the oxide layer as well as by Cu reduction-cum-extrusion from the sulfide layer.

Charging proceeds by a complex route in which initial Li deintercalation is compensated by Mn oxidation prior to the reinsertion of some Cu. As charging continues, Cu insertion fails to fully compensate Li deintercalation, as far as 2.75 V, resulting in manganese and copper oxidation and the formation of a new minority phase with a sharply contracted cell volume, which may be entirely devoid of cations in the sulfide layer and presents a target for bulk chemical synthesis.

The mechanisms of reactions upon discharge and charge are fundamentally different, involving not only different phases but also different oxidation states, resulting in a “path hysteresis” mechanism. The voltage asymmetry observed between discharge and charge during the first cycle can be explained by these differences. Charging to 3.75 V is required to effect almost complete Cu reinsertion and the restoration of a phase similar to Sr_2_MnO_2_Cu_3.5_S_3_. The result is a second discharge profile that mirrors that of the discharge of the pristine material. This reinsertion most likely proceeds through dissolution of Cu into the electrolyte, with loss of these ions being a real possibility. The plating of these ions onto the negative electrode, together with the larger amounts of mass transport and reorganization involved in the cycling at 3.75 V, is likely the culprit of the strong capacity loss observed upon cycling MnCu(II) over this extended voltage window. This work demonstrates the complexity of the displacement reactions in these layered oxysulfides, and the valuable information obtained in this study may provide further insight into other structurally related compounds, regarding their function in the lithium ion batteries.

## Supporting Information Available

The GITT result, cycling curve, Rietveld refinement plots of *in situ* XRD data, and Cu and S K-edge XANES spectra (PDF)
