# Supplementary material for: Structural Evolution of Layered Manganese Oxysulfides during Reversible Electrochemical Lithium Insertion and Copper Extrusion
Source: Chem Mater. 2021 May 24;33(11):3989–4005. doi: 10.1021/acs.chemmater.1c00375 (PMC8276577; doi:10.1021/acs.chemmater.1c00375)
Supplement: Supplementary file 1 — cm1c00375_si_001.pdf [file cm1c00375_si_001.pdf]

SUPPORTING INFORMATION

Structural Evolution of Layered Manganese Oxysulfides during Reversible Electrochemical Lithium Insertion and Copper Extrusion

Sunita Dey,[a] Dongli Zeng,[b] Paul Adamson,[c] Jordi Cabana,[b,d] Sylvio Indris,[b,e] Jingyu Lu,[a] Simon J Clarke, [c,*] and Clare P Grey [a,b,*]

[a]Department of Chemistry, University of Cambridge, Lensfield Road, Cambridge, CB2 1EW U.K.
[b]Department of Chemistry, State University of New York, Stony Brook, NY 11794-3400, U.S.A.
[c]Department of Chemistry, University of Oxford, Inorganic Chemistry Laboratory, South Parks Road, Oxford, OX1 3QR, U.K.
[d]Current address: Department of Chemistry, University of Illinois at Chicago, Chicago, Illinois 60607, USA
[e]Current address: Institute for Applied Materials-Energy Storage Systems, Karlsruhe Institute of Technology, Hermann-von-Helmholtz-Platz 1, 76344 Eggenstein-Leopoldshafen, Germany

* cpg27@cam.ac.uk
* simon.clarke@chem.ox.ac.uk

Table S1. Summary of the sample descriptions during each process.

	Sample label & name	Descriptions
The 1st discharge	a: Li1.0	Partially lithiated sample obtained following insertion of 1.0 mol of Li per formula unit.
	b: Li2.0	Partially lithiated sample obtained following insertion of 2.0 mol of Li per formula unit.
	c: Li3.0	Partially lithiated sample obtained following insertion of 3.0 mol of Li per formula unit.
	d: Li4.0	Sample discharged to 1.1 V. The lithium content for this fully lithiated sample should be around 4.0 mol but may vary slightly from different batteries. It is denoted as “Li4.0” for consistency with other discharged samples during the 1st discharge.
The 1st charge (after the 1st discharge to 1.1 V)	e: Ch1.8V	Sample charged back to 1.8 V after being discharged to 1.1 V during the 1st discharge. It is after the 1st process at ~ 1.8 V of the charge.
	f: Ch2.5V	Sample charged back to 2.5 V after being discharged to 1.1 V during the 1st discharge. It is the onset of the 2nd process at ~ 2.5 V of the charge.
	g: Ch2.75V	Sample charged back to 2.75 V after being discharged to 1.1 V during the 1st discharge. It is after the 2nd process at ~ 2.5 V of the charge.
	h: Ch3.3V	Sample charged back to 3.3 V after being discharged to 1.1 V during the 1st discharge. It is during the 3rd process at ~ 3.3 V of the charge.
	i: Ch3.75V	Sample charged back to 3.75 V after being discharged to 1.1 V during the 1st discharge. It is after the 3rd process at ~ 3.3 V of the charge.
The 2nd discharge (after the 1st charge to 3.75 V)	j: (H)2ndDis_1.5V	Sample at an early stage of the 2nd discharge after the 1st charge to 3.75 V. The Li content in this sample is comparable with that in sample a: Li1.0 during the 1st discharge.
	k: (H)2ndDis_1.1V	Sample at the end of the 2nd discharge (discharged to 1.1 V) after the 1st charge to 3.75 V. This sample resembles to the fully discharged sample d: Li4.0 during the 1st discharge.
The 2nd discharge (after the 1st charge to 2.75 V)	l: (L)2ndDis_1.7V	Sample at a middle stage of the 2nd discharge after the 1st charge to 2.75 V. It is after the 1st process at ~2.2 V during the 2nd discharge.
	m: (L)2ndDis_1.1V	Sample at the end of the 2nd discharge (discharged to 1.1 V) after the 1st charge to 2.75 V.

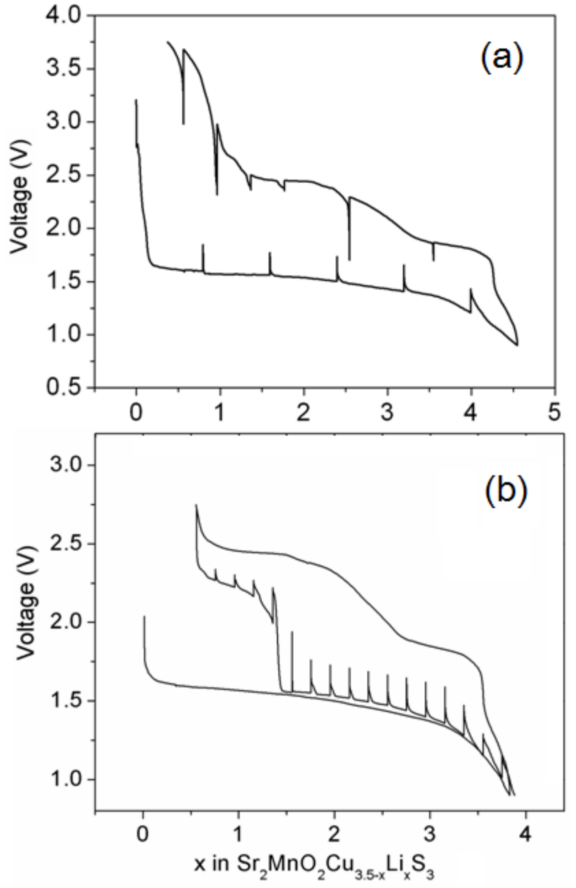

Figure S1. Open circuit voltage curve of MnCu(II)|Li batteries during (a) the 1st cycle, and (b) the 2nd discharge after being charged to 2.75 V, obtained by the galvanostatic intermittent titration technique (GITT). The batteries were both operated at a C/20 rate. For each step, the battery shown in (a) was (dis)charged for 4 h and then allowed to rest for 8 h, and the one shown in (b) was discharged for 1 h and then allowed to rest for 8 h.

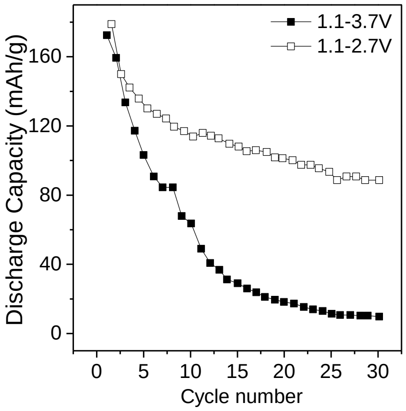

Figure S2. Cycle performance of MnCu(II) using the 1.1 to 3.75 V and 1.1 to 2.7 V windows. The applied current rate is C/20.

Table S2. Refinement results from the *in situ* PXRD measurements for OS-I before and after the 1st discharge and for the OS-II phase after charging to 2.75 V.

Compound	$Sr_2MnO_2Cu_{3.5}Se_3$ OS-I pre-1st discharge. ("Start" in Figure S3)	$Sr_2MnO_2Li_4S_3$ OS-I end of 1st discharge to 1.1 V vs Li^+/Li (point d in Figure S3)	$Sr_2MnO_2Cu_xLi_ySe_3$ OS-II after 1st charge to 2.75 V vs Li^+/Li (point g in Figure S3)
Radiation	PXRD		
Space group	P4/mmm		
a (Å)	4.0193(4)	4.0465(6)	4.0153(8)
c (Å)	11.402(2)	11.777(1)	11.260(6)
c/a	2.8368(6)	2.9104(6)	2.804(2)
Volume (Å^3)	184.20(5)	192.84(6)	181.5(1)
z(Sr)	0.1511(7)	0.1430(9)	0.160(2)
z(S1)	0.261(1)	0.253(2)	0.279(3)
z(Cu/Li(1))	0.358(5)	0.383[a]	0.31(1)
x(Cu(2))	0.391(4)	0.360[a]	0.32(1)
z(Cu(2)	0.410(2)	0.40[a]	0.41(1)
occ Cu/Li(1)	0.24(2) Cu	1.00(2) Li	0.18(2) Cu
occ Cu(2)	0.30(1) Cu	0.00(1) Cu	0.16(1) Cu
Cu / Li per Mn	3.4(1) Cu	4.0(1) Li	2.1(1) Cu[b]
wRp	0.032	0.031	0.030

Coordinates: Sr 2*h* (0.5, 0.5, *z*Sr); Mn 1*a* (0,0,0); O 2*f* (0, 0.5, 0); S(1) 2*g* (0, 0, *z*(S1)); S(2) 1*d* (0.5, 0.5, 0.5); Cu/Li(1) 4*i* (0, 0.5, *z*(Cu/Li(1))); Cu(2) 8*s* (*x*(Cu(2)), 0, *z*(Cu(2)))

[a]not refined.

[b]scattering in the sulfide layer of the OS-II phase modelled as exclusively Cu, but the phase contains some residual Li and vacant sites in the sulfide layer.

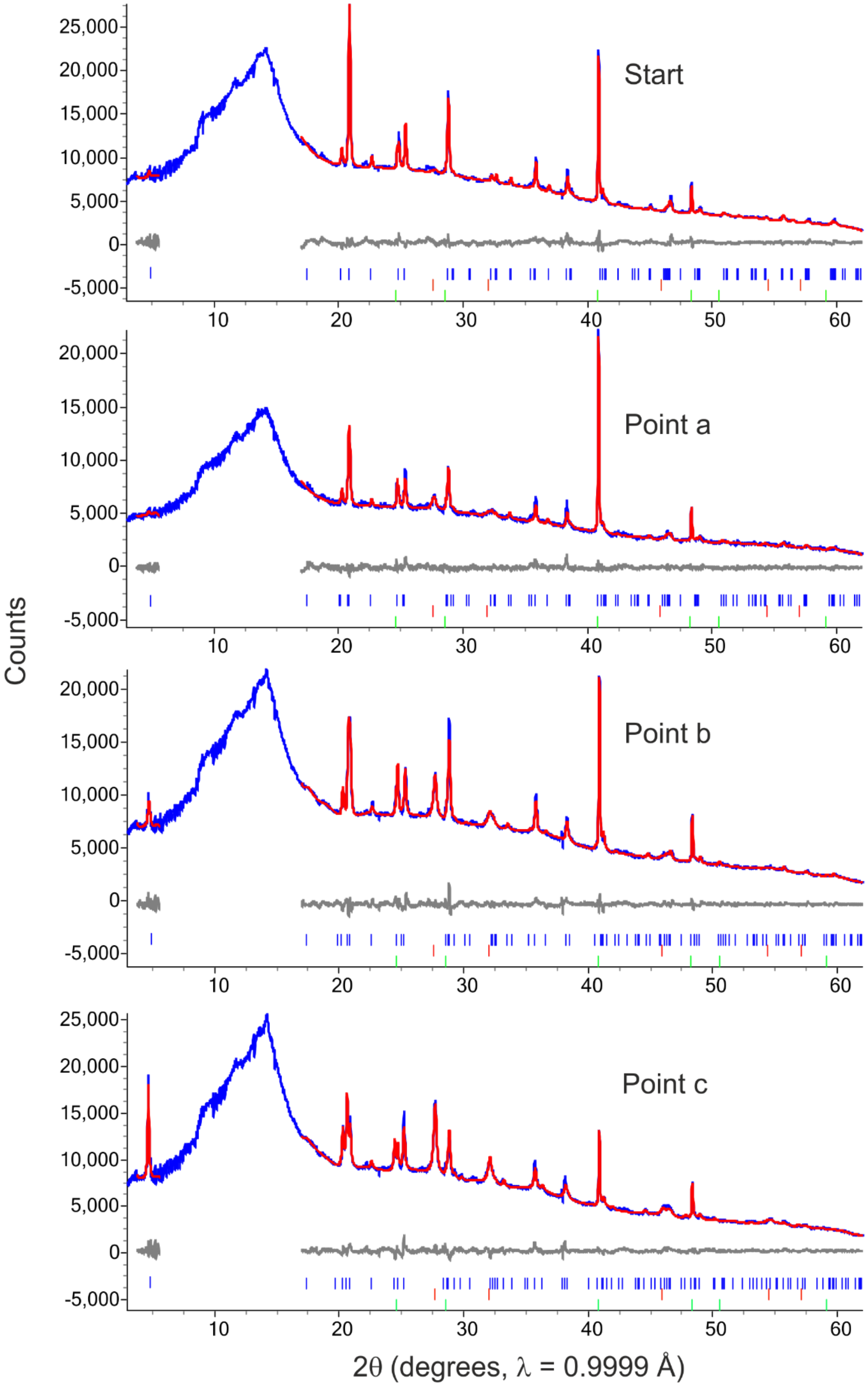
Start
Point a
Point b
Point c
Counts
2θ (degrees, λ = 0.9999 Å)
25,000
20,000
15,000
10,000
5,000
0
-5,000
10
20
30
40
50
60

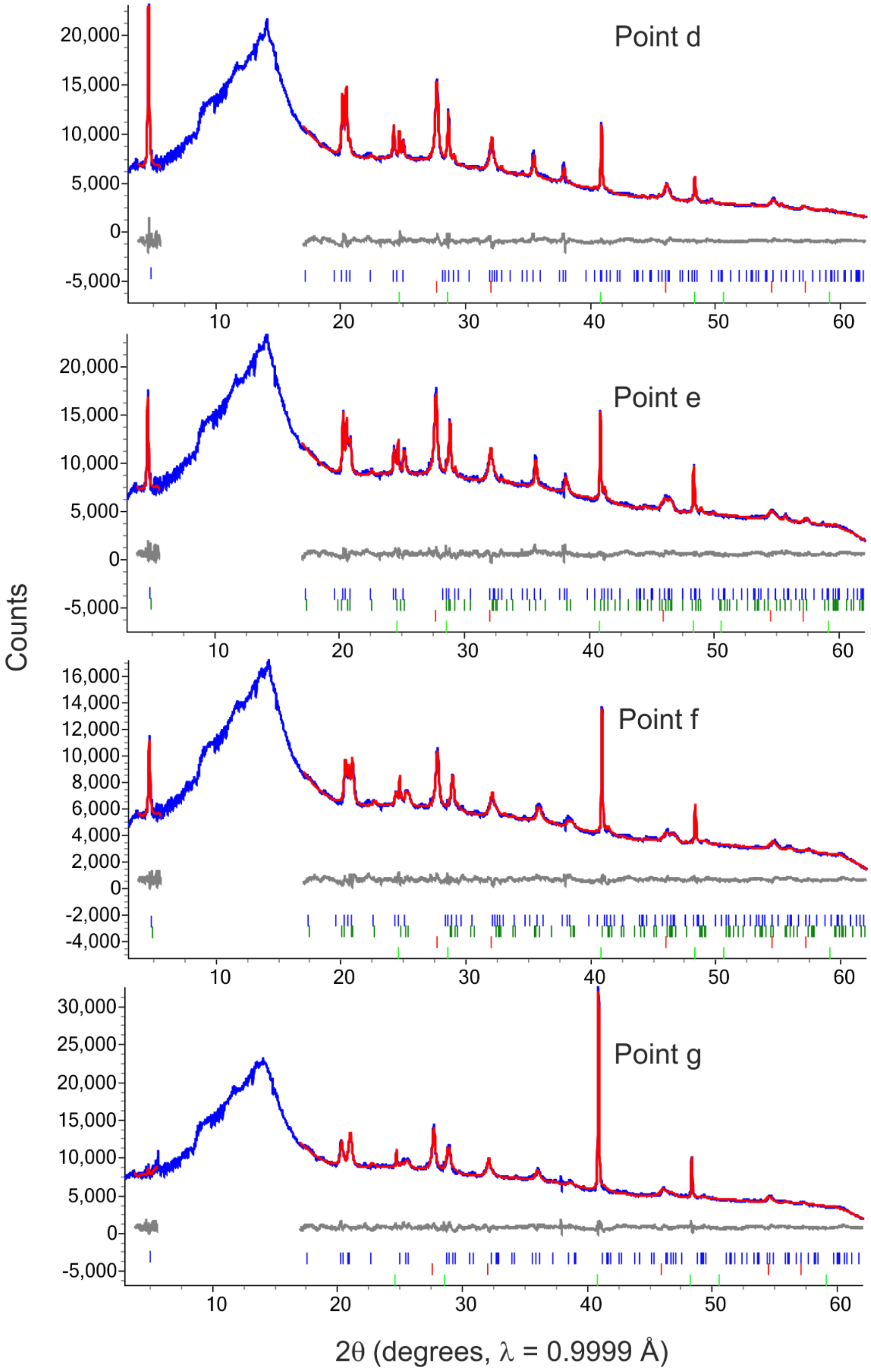

Figure S3. Rietveld refinements against *in situ* X-ray diffraction data. The data at start of the discharge and the points corresponding to the letters in the discharge/charge voltage profiles in the main paper (Figure 2b) are shown from top to bottom. The output parameters from the refinements are shown in Figure 4. The data (blue) calculated (red) and difference (grey) profiles are shown. Tick marks are from bottom: the Al parts of the sample holder (light green), the elemental Cu in the main phase (red) and the two oxysulfide phases (in the two-phase region the tick marks for the OS-I phase are above those of the OS-II phase).

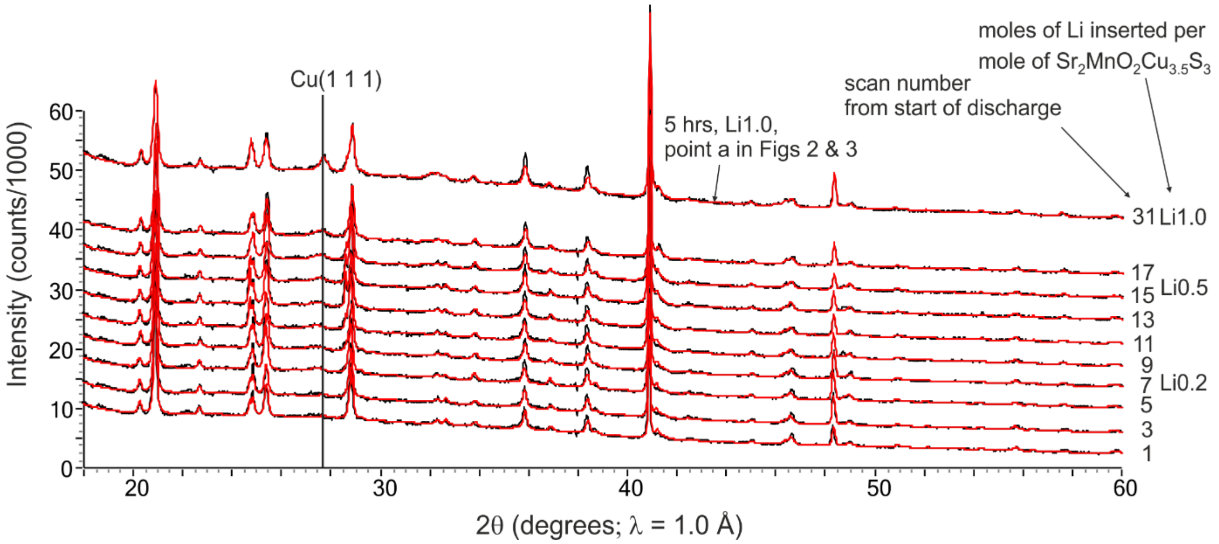

Figure S4. Rietveld refinements against the *in situ* PXRD data collected at the start of the first discharge showing the development of the Cu (111) reflection. The peak is clearly discernable, above the background level of the first scan, in the early scans which correspond to numbers of moles of inserted Li which are much smaller than the number of moles of tetrahedral site vacancies (0.5) per mole of $Sr_2MnO_2Cu_{3.5}S_3$. This shows that the initial stage does not just involve the insertion of 0.5 moles of Li to fill these vacancies, but also involves extrusion of Cu, at least from the point where 0.2 moles of Li have been delivered per mole of $Sr_2MnO_2Cu_{3.5}S_3$. Note that measurements were of poor signal-to-noise ratio between scans 17 and 31 due to experimental problems on the beamline.

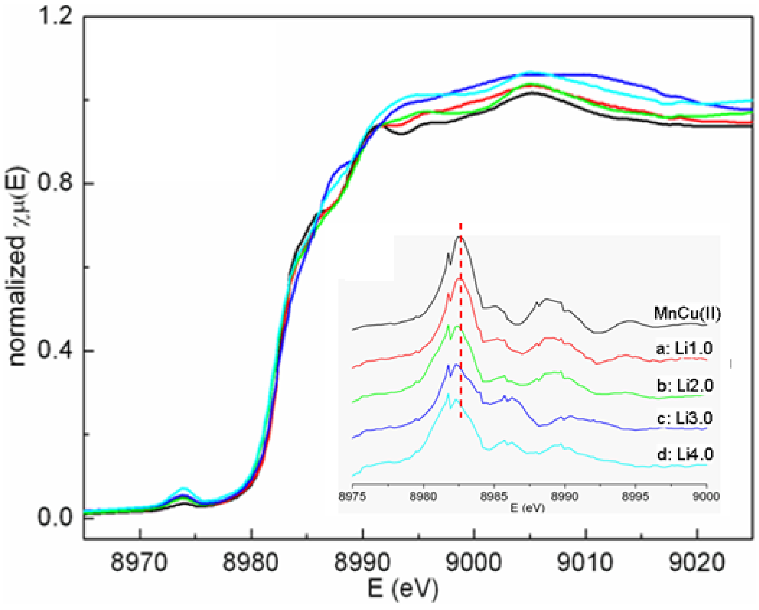

Figure S5. Normalized Cu K-edge XANES spectra of pristine and lithiated MnCu(II) phases during the 1st discharge, and inset stacked plot of the corresponding first derivative curves. The dashed line in (b) indicates the energy position observed for the pristine sample MnCu(II).

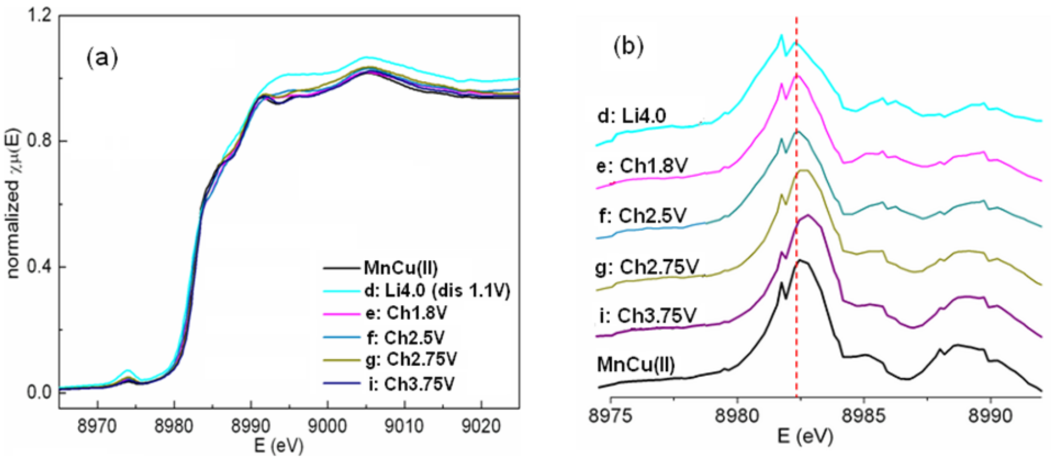

Figure S6. (a) Normalized Cu K-edge XANES spectra of MnCu(II) phases during the 1st charge, and (b) a stacked plot of the corresponding first derivative curves. The spectra of pristine MnCu(II) and the fully lithiated form (d: Li4.0) are also included for comparison. The dashed line in (b) indicates the energy position observed for the fully lithiated phase (d: Li4.0)

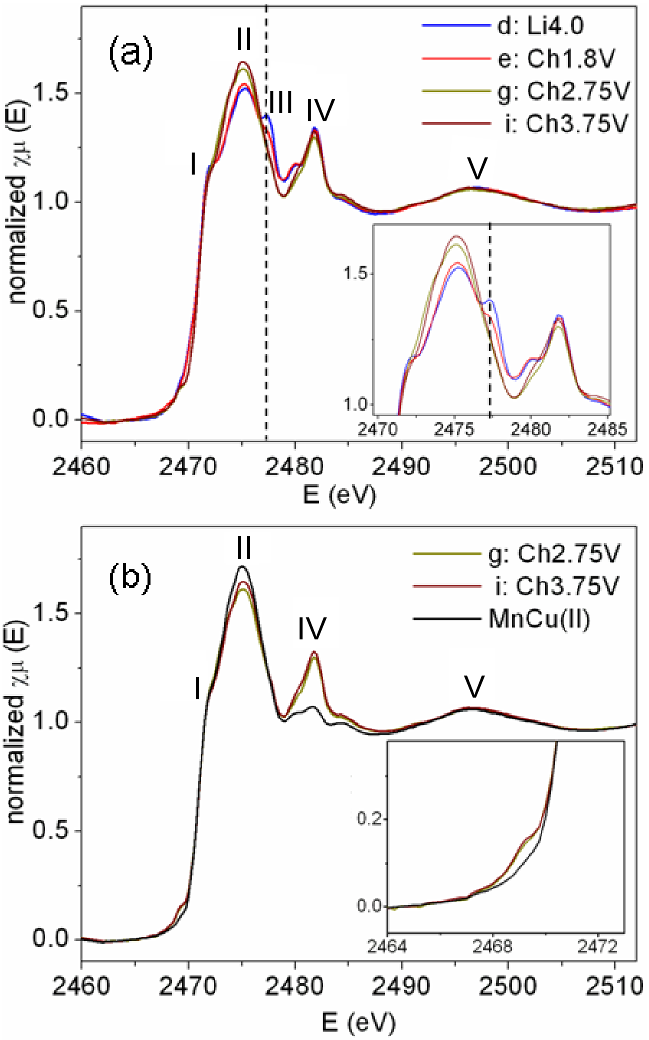

Figure S7. Normalized S K-edge XANES spectra of (a) MnCu(II) phases during the 1st charge and (b) the pristine MnCu(II) and two samples charged to 2.75 and 3.75 V, respectively. The insets show zoomed views of the spectra: (a) between 2470 to 2485 eV, and (b) between 2464 to 2473 eV. The absorption features are labeled with numbers I to V (see text).

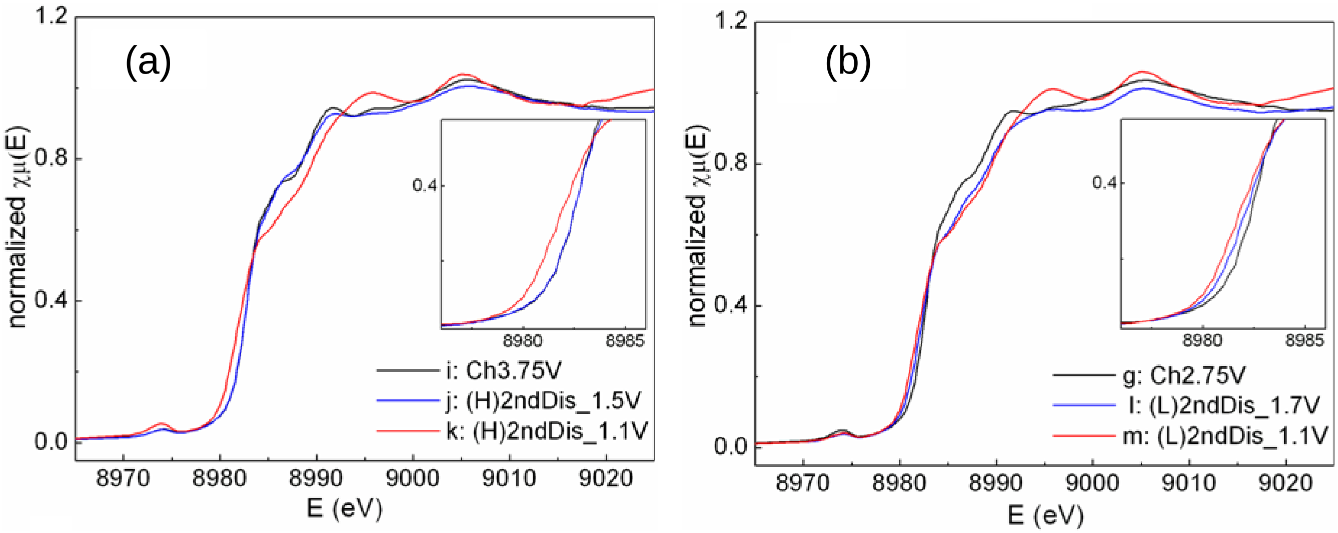

Figure S8. Normalized Cu K-edge XANES spectra of MnCu(II) phases during the two 2nd discharge processes after the 1st charge. The cut off voltage of the 1st charge is (a) 3.75 V, and (b) 2.75 V. The insets in (a) and (b) are zooms of the absorption edge regions.

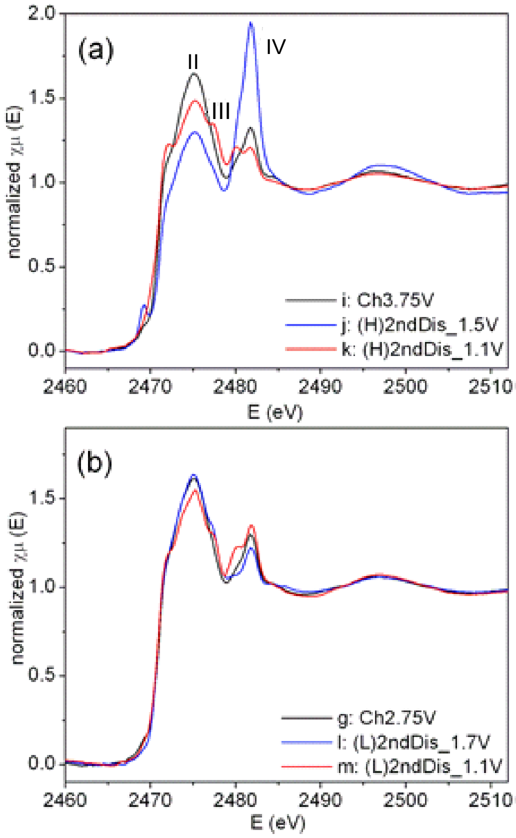

Figure S9. Normalized S K-edge XANES spectra of MnCu(II) phases during the two 2nd discharge processes after the 1st charge. The cut off voltage of the 1st charge is (a) 3.75 V, and (b) 2.75 V, respectively.
